# Multi-oscillation microrheology *via* acoustic force spectroscopy enables frequency-dependent measurements on endothelial cells at high-throughput†

**DOI:** 10.1039/d0lc01135e

**Published:** 2021-03-16

**Authors:** Alfred Nguyen, Matthias Brandt, Till M. Muenker, Timo Betz

**Affiliations:** Institute of Cell Biology, University of Münster Münster Germany a.nguyen@uni-muenster.de; Third Institute of Physics—Biophysics, University of Göttingen Göttingen Germany timo.betz@phys.uni-goettingen.de

## Abstract

Active microrheology is one of the main methods to determine the mechanical properties of cells and tissue, and the modelling of these viscoelastic properties is under heavy debate with many competing approaches. Most experimental methods of active microrheology such as optical tweezers or atomic force microscopy based approaches rely on single cell measurements, and thus suffer from a low throughput. Here, we present a novel method for frequency-dependent microrheology on cells using acoustic forces which allows multiplexed measurements of several cells in parallel. Acoustic force spectroscopy (AFS) is used to generate multi-oscillatory forces in the range of pN–nN on particles attached to primary human umbilical vein endothelial cells (HUVEC) cultivated inside a microfluidic chip. While the AFS was introduced as a single-molecule technique to measure mechanochemical properties of biomolecules, we exploit the AFS to measure the dynamic viscoelastic properties of cells exposed to different conditions, such as flow shear stresses or drug injections. By controlling the force and measuring the position of the particle, the complex shear modulus G*(*ω*) can be measured continuously over several hours. The resulting power-law shear moduli are consistent with fractional viscoelastic models. In our experiments we confirm a decrease in shear modulus after perturbing the actin cytoskeleton *via* cytochalasin B. This effect was reversible after washing out the drug. Additionally, we include critical information for the usage of the new method AFS as a measurement tool showing its capabilities and limitations and we find that for performing viscoelastic measurements with the AFS, a thorough calibration and careful data analysis is crucial, for which we provide protocols and guidelines.

## Abstract 2

We present a novel method for frequency-dependent microrheology on endothelial cell monolayer at high throughput and fluid flow.

## Graphical abstract

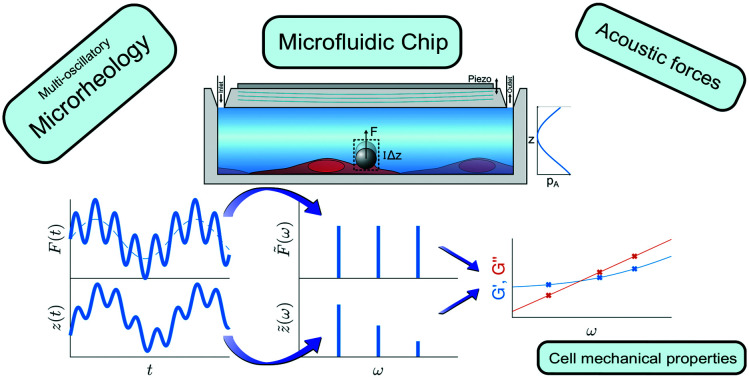

## 1 Introduction

The mechanical properties of cells and tissues are closely related to their biological function, and defects in stiffness and viscosity have been related to several malfunctions and diseases.^1–4^ An example of cells highly exposed to variable mechanical forces are endothelial cells (ECs) which make up the inner wall of blood vessels. They are constantly under mechanical load from the variable shear stresses originating from the blood flow. These forces are known to be important regulators for proper EC function.^5,6^ A highly relevant example are EC stiffness dependent biochemical processes which can promote vascular diseases, such as atherosclerosis.^7,8^ Changes in stiffness between healthy and diseased cells are also often found in other cell types, such as cancer.^3,9^ Despite the importance of a quantitative, mathematical descriptions of cellular viscoelastic properties, a clear model description is not yet available.^10^ In the past, several analogy models for viscoelasticity using springs and dash-pots, like the Maxwell, the Kelvin–Voigt or the standard solid viscoelastic model were introduced,^11,12^ although it became clear more than a decade ago that most cells can be best described by power-law rheology models.^13^ While such competing models are hard to differentiate in the time domain, their differences are quite obvious in the frequency domain, where instead of applying a step force, a well defined oscillatory force is applied. This directly suppresses all noise components that act at different timescales, hence greatly improving signal quality. By testing different frequencies, both the viscoelastic properties can be better defined, and the underlying model better identified. In this work, we use fractional element models to describe our obtained results. These fractional viscoelastic models were recently reviewed by Bonfanti *et al.*^14^

Although it is well known that such frequency-dependent measurements are much more powerful, they have the drawback that a frequency sweep require more experimental time, often lasting several minutes. Classical measurement techniques for such frequency sweeps are optical tweezers^15–18^ and atomic force microscopy.^19–22^ Even though these methods are well established and reliable, they are inherently serial, which means that only a single experiment can be performed at a time. To acquire here significant statistics, multiple single measurements have to be performed. This is a serious drawback, when the temporal changes of a system are of interest. For example, that cells need to be monitored over a long time while changing external conditions. Furthermore, it is difficult to perform some of the measurements in closed microfluidic devices, which are often convenient not only to rapidly change the surrounding environment, but also to apply fluid flows that can mimic the physiological situation.

Hence, there is an urgent need for a method that allows to perform frequency-dependent rheology measurements in parallel and within a microfluidic environment.

In this work, we present a novel method that delivers these requirements by allowing microrheology based on acoustic forces inside a microfluidic chip. This is possible using the recently introduced technique of acoustic force spectroscopy (AFS) where forces are applied in parallel in a large field of view, while the recording of the cellular deformation is parallelized by simple image analysis. The AFS is initially designed to measure mechanochemical properties of biomolecules in a high-throughput manner.^23,24^ Recently, the AFS has been used to measure the static spring constant of red blood cells (RBCs) in the time domain by applying a step force.^25^ RBC stiffness is among the best studied subjects in the field of cell mechanics, and the value is well established, backed by a large body of experiments by different methods like optical tweezers, micropipette aspiration and other techniques, all giving consistent results.^26–29^ Surprisingly, this first application of AFS to cell mechanics reports spring constants that are stiffer than typical literature values.^30^

Despite the elegance of the AFS method, these discrepancies justify further work regarding the calibration and application of the AFS, which is one aim of this work. Briefly, we present a more detailed calibration method which extends previous work by a) using an appropriate viscosity correction, b) taking the position-dependent force into account and c) including the force dependency on the experimental conditions, such as temperature and medium composition, which affects the optimal driving frequency.

As the correct calibration turned out to be an essential, but cumbersome process we report here a detailed calibration procedure to enable reliable microrheological measurements with the AFS. To demonstrate the potential of the method, we show in the second part of the paper the first frequency-dependent measurements of a monolayer of primary human umbilical vein endothelial cells (HUVEC). Our results of the static elasticity modulus are in perfect agreement with literature values. By exploiting the AFS capacity to perform multi oscillation microrheology over a long time-span, we could show that the previous model using single power-laws are not sufficient to describe the viscoelastic behavior of cells, but that another established fractional element model called generalized Kelvin Voigt model can describe the data best. Moreover, it allows a continuous measurement under a controlled fluid flow enabling the investigation of the viscoelastic properties over time upon variable conditions.

## 2 Materials and methods

### Acoustic force spectroscopy

The acoustic force spectroscopy (AFS; LUMICKS B.V, Netherlands) equipment consists of a Generation 2 AFS module containing a function generator and a temperature controller. Visualization is achieved by an inverted microscope setup with a motorized 10× objective (Nikon, Plan Fluor) for a nanometer-precise *z*-translation, an LED and a uEye camera (UI324xML-M) capable of imaging 1280 × 1024 pixels corresponding to a 678.40 μm × 542.72 μm field of view with a sampling frequency of 59 Hz. The Generation 2 chips consist of a flow chamber with a fill volume of about 6 μl and a piezoelectric element (piezo) on the top of the glass chip to transduce the acoustic waves.^23^ Acoustic forces are applied on 10 μm diameter polystyrene beads (micromer, micromod Partikeltechnologie GmbH). During the experiment the beads are tracked by image recording using the LabVIEW software provided by the manufacturer (LUMICKS B.V) which we have modified for our needs, such as generating oscillatory forces. The *z*-position of each bead is determined using a predefined look-up-table (LUT) ranging from 0 nm to 20 000 nm in 100 nm steps according to van Loenhout *et al.*^31^ Real time image processing allows a live-view of the bead position. The software also generates the voltage signals for the piezo to apply the acoustic pressure which results in a force on the beads. The modified software can generate signals to exert custom, time-varying force profiles, such as force oscillations, and is compatible with the self-written MATLAB (The MathWorks, Inc.) analysis program Kitsune (available on github https://github.com/A141-User/Acoustic-Kitsune) enabling a live-view of the recorded data which can be analyzed afterwards.

### Stokes force calibration of AFS chips

As the software generates voltage signals, a force calibration is required. Stokes force calibration (SFC) yields the conversion factor from voltage to force for the 10 μm-diameter polystyrene beads. Fluorescent (FluoSpheres™, F8834, Thermo Fisher Scientific) and carboxylated beads (micromer, micromod Partikeltechnologie GmbH) are used in culture medium (ECGM/M199 mixture) and water, respectively. Beads are suspended to a concentration of approximately 1 mg ml^−1^ and injected in the chip. This concentration avoids bead clustering and high bead density which would prevent tracking. Temperature is set using the LabVIEW software and the feedback-controlled temperature sensor mounted on the chip. For each bead inside the field of view (FoV) a look-up-table (LUT) between each bead's radial intensity profile and its *z*-position is generated. To displace the beads from the chip surface at least three different acoustic amplitudes (in %) are applied for 1 s. The time between applying these amplitudes is 15 s and 11 s for the SFC in ECGM/M199 and in water, respectively. Typically, about 25 beads at random positions are measured simultaneously inside a FoV. This procedure is repeated after flushing new beads in the FoV. As we find that the conversion factor depends on the position in the FoV, a spatial calibration map is generated by measuring at least more than 1000 beads with well-distributed data points. In case of problematic calibration, such as beads binding unspecifically to the glass surface or tracking errors due to bead clustering, these beads are filtered out before generating the spatial map. A protocol of the SFC and more details on data analysis of the SFC are given in the ESI.† To measure the force dependencies on optimal frequency, temperature and medium, several spatial calibration maps are measured. Each data set includes at least 1000 analyzed beads subsampled into three random groups and the statistical significant difference between the respective condition groups is determined by a one-way ANOVA, while a two-sample *t*-test is performed to check the significance of two neighbouring data sets.

### Determination of the bead immersion half-angle

**Fig. 1:**
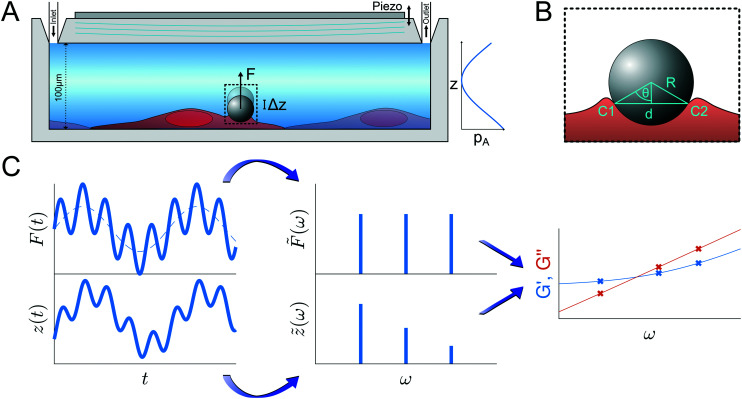
Principle of the microrheological experiment using the AFS. (A) Schematic cross-section of the AFS showing the fluid chamber with a cell monolayer. The oscillating piezoelectric element generates a standing pressure wave *p*_A_ inside the fluid chamber resulting in a force in the *z*-direction on a small bead attached on the cell. The height of the chamber is approximately 100 μm and the width 2 mm. (B) Illustration of a partially immersed bead on a cell surface. Bead and cell surface come in contact at points C1 and C2. The distance between C1 and C2 represents the diameter *d* of the bead-cell contact circle, *R* denotes the bead radius and *θ* the immersion half-angle. (C) Analysis of the multi-oscillation microrheology. The force is modulated with multiple superimposed frequencies and the measured *z*-position follows with the same frequencies. The Fourier transform *F̃*(*ω*) and *z̃*(*ω*) of the force and the *z*-position is calculated. The resulting complex shear modulus is then obtained with eqn (4).

Human umbilical vein endothelial cells (HUVECs) are seeded overnight on a 35 mm diameter glass bottom dish (Greiner Bio CELLVIEW Cell Culture Dish) until they form a confluent monolayer. The glass surface has been coated with 68 μg ml^−1^ collagen type I from rat tail (Corning) dissolved in 0.02 M acetic acid for 1 h. In this protocol, only collagen monomers will cover the surface and no hydrogel is polymerized. 10 μm diameter collagen coated polystyrene beads (micromer, micromod Partikeltechnologie GmbH, Germany) are added on top of the HUVEC monolayer and are allowed to sink for 30 min. During the same time period the cell membrane is stained using a plasma membrane dye (CellMask™ Deep Red, ThermoFisher Scientific) at 0.5 μM concentration. 3D-stacks of 90 *Z*-planes à 0.2 μm distance for five different fields of view are acquired with a scientific CMOS camera (Prime BSI Photometrics) using spinning disk confocal microscopy (Nikon Eclipse Ti-E, equipped with CSU-W1 Yokogawa SD head) using a 60× Plan Apo water-immersion objective with a numerical aperture of 1.27 and an excitation laser of 640 nm wavelength. Using a custom MATLAB code the cell surface was determined by locally fitting polynomials to the fluorescence intensity data. For a total of 43 beads the diameter *d* (see Fig. 1B) of the bead-cell contact circle was determined averaging over 4 different contact points chosen by eye. Knowing the bead radius *R* the immersion half-angle *θ* was calculated as1
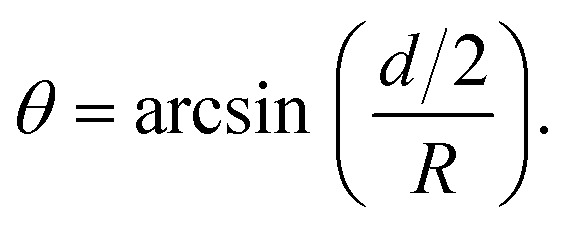


### Microrheological experiment

The principles of the microrheological experiment are shown in Fig. 1. The chip with the HUVEC monolayer is placed on the AFS microscope setup and temperature-controlled to 36 °C ± 1 °C to avoid temperatures above 37 °C given the uncertainty of the heating system. The connected syringe pump is kept at a flow rate of 1.66 μl min^−1^. Through an injection site 10 μm collagen coated polystyrene beads (micromer, micromod Partikeltechnologie GmbH) suspended in CO_2_-charged medium are inserted using a syringe. After closing the outlet valve to prevent flow during the bead incubation time (about 5 min) the valve is reopened and the resulting flow removes unattached beads. This process is repeated until a desired amount of beads has attached to the monolayer inside the calibrated field of view. Then, the flow is stopped for 5 min and the temperature is decreased to approximately 33 °C as the acoustic force application increases the temperature inside the chip. After generating the look-up-table (LUT) for each bead in the FoV, the acoustic forces inside the AFS chip are applied, pulling the beads upwards, away from the surface. For the multi-frequency oscillatory (mOsc) force the frequencies 0.1 Hz, 0.5 Hz and 1.5 Hz are applied simultaneously to span more than one order of magnitude in a single measurement. The choice of these frequencies are mainly related to the hardware. Choosing other or more frequencies is possible. Higher frequencies would require a faster camera for reliable bead tracking. Continuous mOsc forces are exerted on the beads attached on the cells of the monolayer for about 3700 s using the following protocol for drug treatment and control conditions:

a) for 1000s a flow rate 1.66 μl min^−1^ of CO_2_-charged medium is applied to mimic the culture conditions;

b) change of medium containing syringe to apply control (CO_2_-charged medium) or treatment (1 μg ml^−1^ cytochalasin B, Sigma-Aldrich) medium for 1000 s at a flow rate of 30 μl min^−1^. As the volume to be filled is about 300 μl the new conditioned medium arrives at the chamber after 600 s;

c) finally, the flow rate is decreased to the initial 1.66 μl min^−1^ for 1700 s. The syringe exchange takes less than 100 s, and during this change the flow rate is initially lower and then higher when adjusting the pusher block of the syringe pump to the plunger of the syringe.

After this protocol the mOsc force is stopped and the flow rate is shortly increased to 30 μl min^−1^ to remove detached beads and then the temperature is set back to 36 °C and the flow rate to 1.66 μl min^−1^.

To confirm cell recovery after cytochalasin B (cyto B) treatment, the mOsc force measurement is not stopped after c), but the syringes are exchanged back to the CO_2_-charged medium at a flow rate of 30 μl min^−1^ for 1100 s. Then, the flow is decreased again to 1.66 μl min^−1^ until the end of the measurement.

### Cell culture

Human umbilical vein endothelial cells (HUVECs) are kindly provided by Prof. Dr. V. Gerke (Institute of Medical Biochemistry, University of Münster, Münster, Germany) and are obtained according to the protocol described in Jaffe *et al.*^32,33^ HUVECs are cultivated in a 60 mm × 15 mm culture dish (Corning Cell Culture Dish, Corning CellBIND Surface, nonpyrogenic, polystyrene) at 37 °C and 5% CO_2_ in a humidified incubator. Culture medium consists of a 50/50 mixture of two medium solutions; namely, endothelial cell growth medium 2 (ECGM; PromoCell, Germany) supplemented with the SupplementMix for ECGM 2 (PromoCell), and Medium 199 Earle's (F0615, Biochrom GmbH, Germany or M2154, Sigma-Aldrich) with 2.2 g l^−1^ NaHCO_3_, without l-glutamine, supplemented with 10% fetal bovine serum (F7524, Sigma-Aldrich), 30 μg ml^−1^ gentamicin (Gibco), 0.015 μg ml^−1^ amphotericin B (Gibco) and 0.2 units per ml heparin (H3149, Sigma-Aldrich). For flow experiments, CO_2_-charged medium was used, which refers to the described cell culture medium, which is pH-stabilized by equilibration in a 5% CO_2_ atmosphere. HUVECs of passages 3–6 are split at 70–90% confluence, and are discarded after passage 6. To allow cell spreading, the flow chamber of the AFS chip is coated with 68 μg ml^−1^ monomeric collagen type I from rat tail (Corning) dissolved in 0.02 M acetic acid for at least 4 h at room temperature. The incubation time is chosen such that it works well within the experimental procedure. The thickness of collagen coating is expected to be in the low nm range and the stiffness comparable to the glass. HUVECs are seeded onto the flow chamber of the AFS chip at a density of about 2 × 10^6^ cells per ml before placed into a dry incubator at 37 °C and 5% CO_2_. For long term visualization of cells, the chip is mounted on the AFS equipment and heating is ensured by the temperature controller of the instrument. After >2 h most of the cells have attached onto the surface. Using a syringe pump (AL-2000, World Precision Instruments) CO_2_-charged medium is pumped through the flow chamber at a flow rate of 500 μl min^−1^ for <1 min to flush out non-attached and possible dead cells. Then, the flow rate is set to 1.66 μl min^−1^ to renew the medium during cultivation. For the monolayer experiments, HUVECs are grown inside the incubator for 70–100 h.

### Data analysis

#### Power-law rheology models

As we observe power-law behavior for the viscoelastic shear modulus, recent fractional models are considered to explain the data.^14^ A detailed explanation of the models is found in the ESI.† Briefly, we apply either a single fractional element model (SFE) or a generalized Kelvin–Voigt model (GKV) that is based on a combination of fractional elements.^34^ In the SFE model, the complex shear modulus is:2
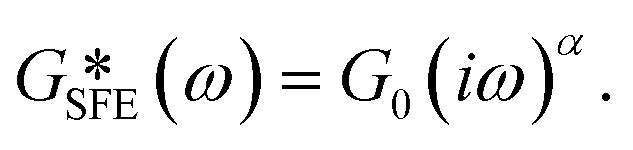
yielding a power-law behavior as often^35,36^ used to describe linear viscoelastic materials.

The second model is a combination of two SFEs resulting in the simplified GKV model:3
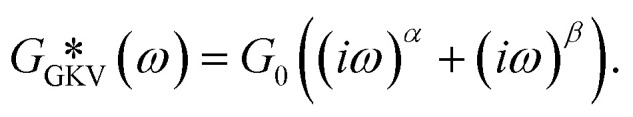
The expressions of the real and imaginary part of eqn (3) are shown in the Appendix (eqn (12) and (13)). *G*_0_ can be interpreted as an apparent shear modulus or as the steady state elasticity comparable to the Young's modulus *E* for *β* = 0 by *E* = 2*G*_0_(1 + *ν*) with the Poisson's ratio *ν*. The two power exponents (*α*, *β*) define a crossover frequency *ω*_*x*_ (eqn (14) in the Appendix) that shows the transition of solid-like state to fluid-like or *vice versa* of the material.

To obtain the stress *σ* and the strain *ε* from the measured force and bead displacement *z* we follow the approach previously developed for an optical tweezers experiment.^36,37^ The resulting complex modulus is calculated depending on the immersion of a bead inside the material characterized by an immersion half-angle *θ*, see Fig. 1B, *i.e.*4
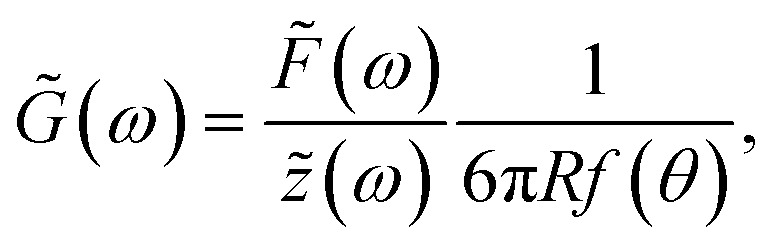
5
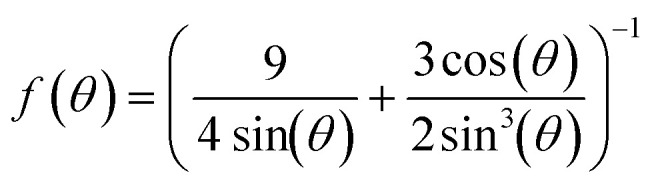
With [*f*(*θ*) < 1, ∀ *θ* ∈ (10°, 90°)], see ESI† Fig. S12, which will be used for the analysis. For a bead immersed in an infinite medium,^36,38^ the complex modulus would be 
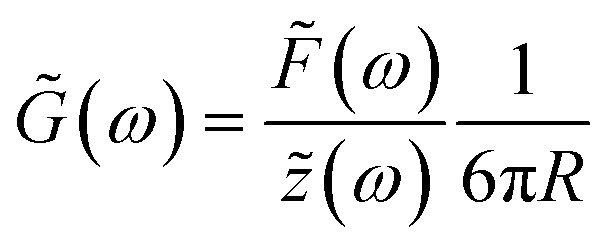
, which is identical to the generalized Stokes–Einstein relation.

## 3 Results

The two main results of this work are, firstly, to provide a clear description for the calibration of the AFS for microrheology, and secondly, to perform the first frequency-dependent microrheology on an endothelial cell monolayer using the AFS. For successful microrheology experiments we found that special care needs to be taken for the bead location, the resonance frequency at the precise temperature and the details of the medium used. To get from the forces to the shear modulus we also determine the bead immersion. Only when these points are well controlled, the microrheology experiments using the AFS yield reliable results.

### Stokes force calibration of AFS chips

Knowledge of the exerted acoustic force inside the AFS chip is essential to use the AFS as a measurement tool. Previous calibration strategies^25^ used the fact that in an overdamped system, the velocity of a particle moving in a liquid of known viscosity depends on the forces acting on it. By observing the particle motion after switching on the pulling force exerted by the AFS, a force profile was calculated. As during this measurement the bead was moving quickly out of focus, the method was termed ‘shooting beads’ method. Intrigued by the discrepancy between the elasticity values reported by this method and the known literature values, we sought to critically investigate the calibration process to identify potential problems and to improve the calibration for the AFS. Although, the method should be in itself robust, as it relies on fundamental physics like the force equilibrium and the well known relation of viscous forces, it turns out that the viscosity correction is a further critical factor to consider.

Briefly, the force *F*_ac_ acting on the particle is expressed by *F*_ac_ = *F*_eff.grav._ + *F*_Stokes_, where *F*_eff.grav._ is the force of gravity and *F*_ac_ is the acoustic force applied by the AFS, which has been well studied and described previously (see Sitters *et al.*^23^ and ESI† for details). *F*_Stokes_ is the viscous force described by Stokes law of viscosity, which was introduced by Stokes in 1851:^39^

6 *F*_Stokes_ = 6π*Rvη*_eff_

where *R* is the particle radius, *ν* its velocity and *η*_eff_ the effective dynamic viscosity of the surrounding fluid. The Stokes force is only valid for particles positioned far away from any wall. Therefore, its application for situations close to boundaries must include a correction factor for the effective viscosity *η*_eff_ = *λη*_fluid_, where *λ* represents a situation-dependent correction factor. As the viscosity is temperature-dependent it can be approximated for water with a modified Andrade equation,^40^ see Appendix (eqn (17)). The viscosity of cell culture medium containing serum is approximated to be the same as water. This is justified by measurements showing that the presence of serum only increases the viscosity by about 5%.^41^

**Fig. 2:**
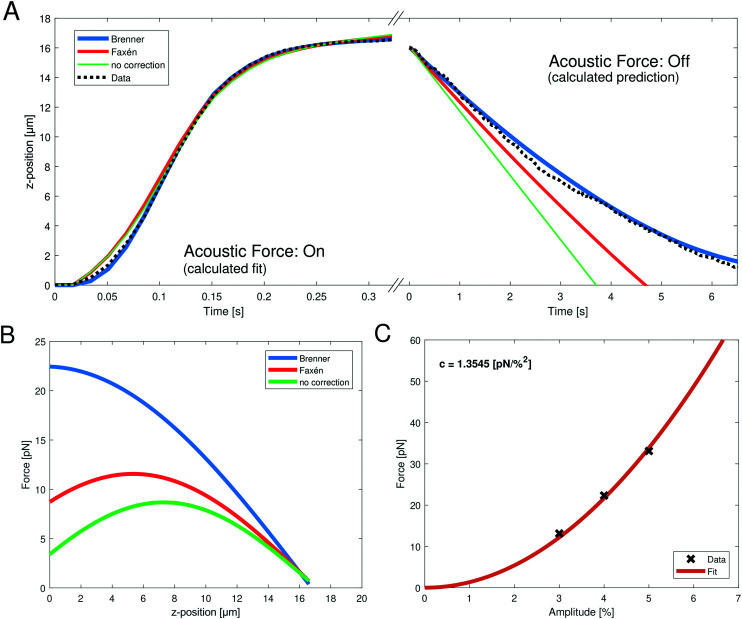
(A, left) Representative, measured *z*-position data (black dotted line) obtained when turning on the acoustic force during the SFC measurement; the bead moves upward towards the acoustic node. The fits can be obtained by integrating eqn (10) with the different viscosity corrections. The goodness of fit is *R*^2^ > 0.9971 for all shown fits (blue: Brenner, red: Faxén, green: no correction). (A, right) The following obtained *z*-position data (black dotted line) after turning off the acoustic force; the bead falls to the ground. Only the gravitational force is relevant and the *z*-position can be calculated using different viscosity corrections (blue: Brenner, red: Faxén, green: no correction). (B) Resulting force profile obtained from the fit parameters with eqn (8) of the positional data from (A) with the different viscosity corrections. The fit parameter values are shown in Table 1. The applied amplitude was 4%. (C) Parabolic fit (eqn (11), orange line) between the acoustic force and the applied amplitude. The force was calculated with the Brenner viscosity correction (eqn (7)) and the data points (black crosses) represent the force at *z* = 1 μm to obtain the conversion factor *c*.

To obtain knowledge about the applied forces a calibration is required that connects the force to the applied acoustic intensity which is controlled by an amplitude value in percentage [%] units. The previously introduced calibration model^24,25^ used a viscosity correction factor *λ* based on Faxén's law (see Appendix, eqn (19)), that is successfully used to calibrate optical tweezers where particles are displaced parallel to the boundary surface. However, as in AFS experiments the bead moves perpendicular to the surface Faxén's law is not suitable (see Fig. 2), hence the previous calibration method can be improved by a more realistic viscosity correction.

Here, we present another viscosity correction factor derived by Brenner^42^ for motion perpendicular to the surface that leads to excellent agreement between predicted bead motion and the calibration measurements.

To differentiate our method from the previous “shooting bead” method, we coined it Stokes force calibration (SFC). It not only integrates the correct viscosity correction, but also includes spatial heterogeneity, temperature and medium composition effect, as described in the following.

Instead of Faxén's law, the viscosity correction for perpendicular translation as given by Brenner^42^ is used:7
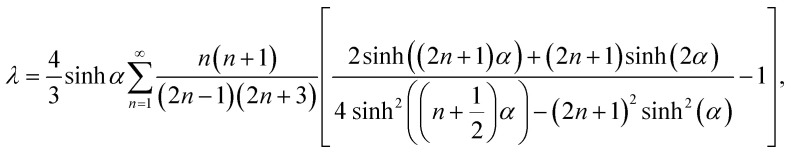
with 
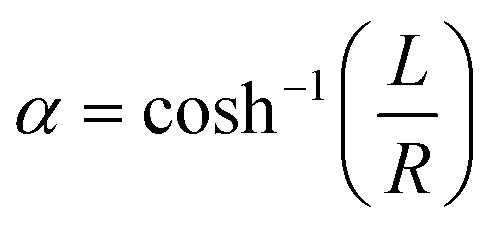
 and *L* the distance of the center of the bead to the surface, *i.e. L* = *z* + *R*. For the numerical computation the sum goes from 1 to 100. From eqn (S9) in the ESI† the acoustic forces can be approximated by:

8 *F*_ac_ ≈ *f*_0_*k*_p_ sin(2(*k*_p_*z* + *ϕ*_p_)),

9
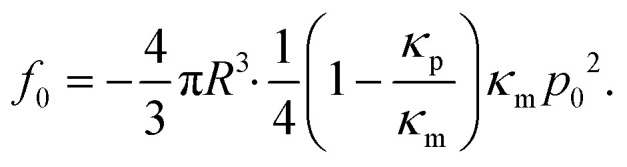
Here *p*_0_ denotes the pressure amplitude, *k*_p_ and *ϕ*_p_ the wave number and the phase of the acoustic wave, respectively. Using the invoked force equilibrium and eqn (8) the *z*-position can be obtained by numerically integrating the velocity10
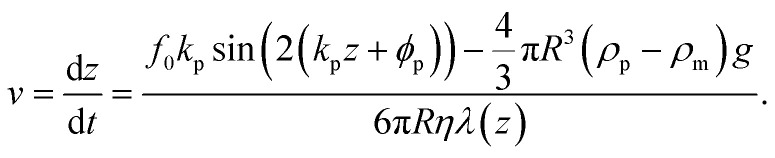
For the calibration, the fit parameters *f*_0_, *k*_p_ and *ϕ*_p_ are optimized until the predicted *z*_num_ and measured *z*_meas_ positions during the force application match best. Thus, neither the knowledge of the compressibility of the particle *κ*_p_ nor of the medium *κ*_m_ is required for the SFC.

Despite theoretical arguments we wondered if we can see experimental evidence supporting the Brenner approach. Therefore, we directly compared the fit quality using no correction, Faxén's law (eqn (19)) and Brenner's solution (eqn (7)). Fig. 2A shows the data and the different fits of the *z*-position during the acoustic force application, and during the gravitational fall of the bead after switching off the acoustic force. While for the upward movement of the bead, all three corrections show good fit qualities (Fig. 2A and Table 1), the problem becomes evident when switching off the AFS, and observing the return of the bead to the glass surface. Here, the only force driving the bead motion is gravity. The right panel in Fig. 2A shows the calculated *z*-position when using different corrections. Here nothing is fitted, but simply the known force of gravity is used. For this test, the impact of the choice for the viscosity correction becomes evident. It clearly shows that using no correction or Faxén's law the measured *z*-position cannot be described. However, using the Brenner correction the *z*-position can be predicted accurately. This is quantified by the excellent *R*^2^ value of the Brenner solution (see Table 1). In a control analysis, where the bead density is used as a fit parameter, the Brenner solution also yields a density that best matches the literature value for polystyrene of 1050 kg m^−3^, further supporting that the Brenner solution is to be used, see ESI† Fig. S2 and Table 1. As the previous calibration in the “shooting bead” method only used the upward motion (*i.e.* acoustic force: on) it is not surprising that the problem was not obvious since it is possible (albeit incorrect) to use Faxén's law to obtain reasonable fit qualities.

**Table 1:** Values of the fit parameters obtained by the SFC to calculate the force profile and the *R*^2^ representing the goodness of fit obtained by the SFC and the calculation of the bead fall shown in Fig. 2A

Correction	*f*_0_ [pN μm]	*k*_p_ [1/μm]	*ϕ*_p_ [rad]	*ρ*_p_ [kg m^−3^]	*R*_on_^2^	*R*_off_^2^
Brenner	488.189	0.046	0.799	1051.55	0.9997	0.994
Faxén	172.300	0.067	0.427	1034.52	0.9971	0.176
No correction	108.433	0.080	0.202	1023.85	0.9977	−3.190

Next, we were wondering if the choice of correction factors has an influence on the predicted calibration. Using the obtained fit parameters to calculate the acoustic force profile in the *z*-dimension with eqn (8) it becomes evident that the predicted forces are drastically different (Fig. 2B), especially in the relevant region close to the surface (small *z* values). For the analytic Brenner correction (eqn (7)) the maximum is at the surface of the chip, as expected, which means that it can be approximated as constant close to the chip surface. In contrast, for Faxén's law and without correction the maximum is at a larger distance of *z* > 5 μm from the surface. Furthermore, the absolute value of the prefactor *f*_0_ which yields information of the force varies by almost a factor of five depending on the analysis (see Table 1). This has profound effects on the final quantitative measurements, and may partially explain the discrepancy between previous microrheology measurements on RBCs and literature values.

After providing experimental evidence justifying the Brenner solution as viscosity correction, we obtained acoustic forces at different amplitudes. The relation between the force *F*_ac_ and the amplitude *V*_%_ is quadratic

11 *F*_ac_ = *cV*_%_^2^

with the conversion factor *c* in [pN %^−2^] because the amplitude is directly proportional to the pressure. The conversion factor is obtained by fitting a polynomial function of second order (eqn (11)) to at least two different, measured force values at a non-zero amplitude (*V*_%_ ≠ 0%), see Fig. 2C. Indeed, the quadratic relation can be seen in the measured data. The force values are obtained from the force profiles at *z* = 1 μm.

### AFS requires position-dependent calibration

After having identified the correct viscosity correction for the AFS calibration using SFC we turned to the lateral distribution of the calibration factors. This is motivated by the fact that only in an ideal infinite system a homogeneous pressure field is expected. Experimentally, the chip is constrained by walls, which also results in low lateral forces that can be clearly observed when unbound beads gather in the first node of the acoustic pressure field in the *z*-direction. In this plane even small lateral forces lead to drift and accumulation of forces in a stripe pattern. The lateral forces are in the order 0.1 pN, and hence several orders of magnitude smaller than the *z*-forces (Fig. 2C). Nevertheless, their presence suggests that the forces are position-dependent. To determine a possible lateral force inhomogeneity, we calibrated multiple beads at different positions inside the full field of view (FoV) to obtain a spatial map of calibration values.

**Fig. 3:**
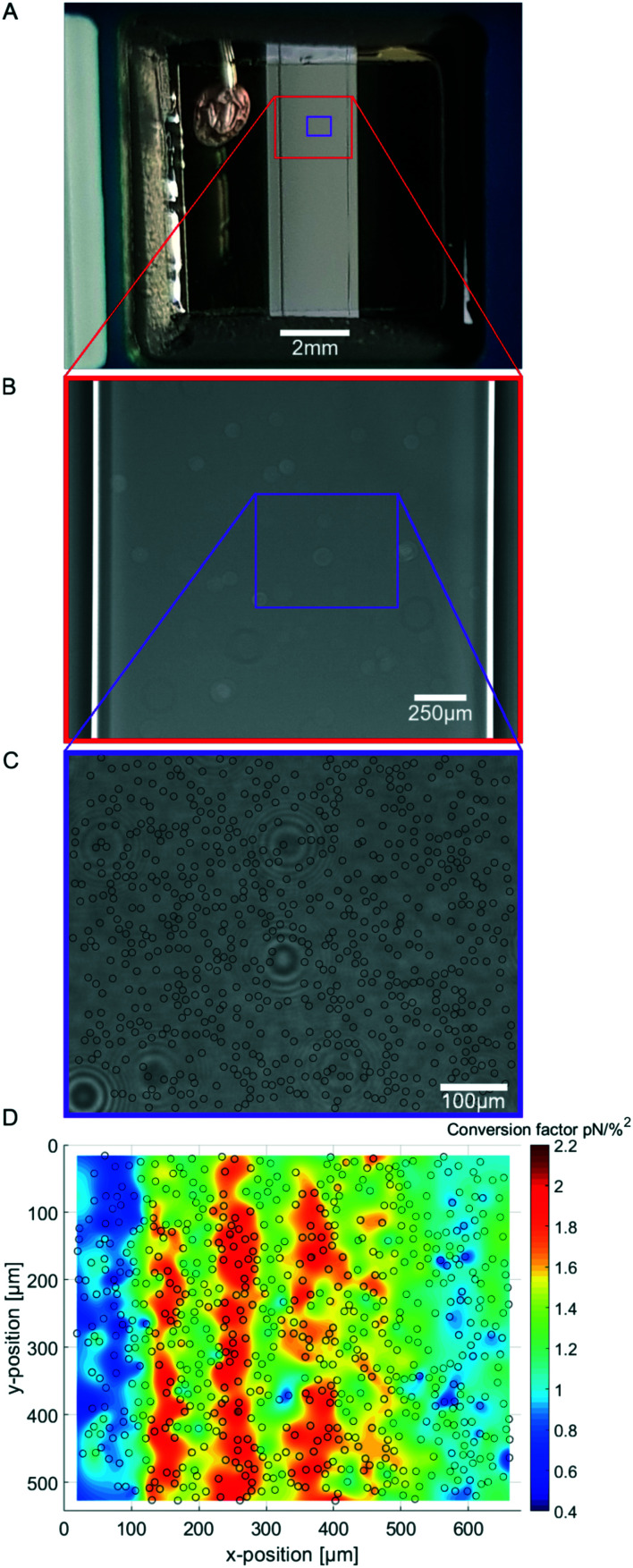
(A) Image of the AFS chip showing the window where the flow chamber and the transparent, quadratic piezoelement can be seen. The connection to the signal generator is placed on the top-left side. (B) Magnified image of the flow chamber showing the flow chamber borders. (C) Further magnified image of the flow chamber showing a typical field of view for the experiments including black circles marking the bead locations for the calibration map. The random dirt on the piezo is clearly visible. (D) Spatial calibration map (*n* = 1190 beads, merged to 706 beads) obtained by performing the SFC and analyzing with our software Kitsune.

To visually compare the sizes of the chamber and FoV, the spatial dimension of the flow chamber underneath the transparent piezo is shown in Fig. 3A and B and the typical field of view with a cumulative indication of the calibrated beads is shown in Fig. 3C. The spatial calibration map representing the conversion factor *c* at different lateral positions is obtained by performing the SFC on over 1000 randomly distributed beads inside the same field of view (FoV) shown in Fig. 3D. As suspected, the force calibration is highly dependent on the lateral positions. The calibration map (Fig. 3D) shows the resulting force distribution in the chip which varies up to a factor of six depending on the position of the bead. Surprisingly, the most efficient force generation is not directly at the center of the chip in the short axis (*x*-position), but slightly shifted outwards. In fact, typically the change of the force is even very high close to the center; in the shown FoV (Fig. 3C) the center of the flow chamber is at around 100 μM. Only about a quarter of the short axis of the flow chamber has an area of high forces while in other FoVs there are very low forces which may render them unusable. In our hands, we found that every chip is individual and therefore differs in force and force distribution. This may be due to the coupling to the piezo and due to the geometry of the chip layers which is important for the force generation. Hence, to perform reliable quantitative analysis it is very important to determine the spatial calibration map for every chip and FoV to be used in the experiments.

Besides the viscosity calibration, our SFC method improves the calibration for the AFS by additionally taking the spatial contribution into account.

### Forces depend on optimal frequency, temperature and medium

After establishing that the acoustic force is position-dependent, we wondered if further parameters, such as temperature and medium composition might affect the calibration. Therefore, we investigated if the optimal driving frequency for the acoustic field depends on these parameters.

**Fig. 4:**
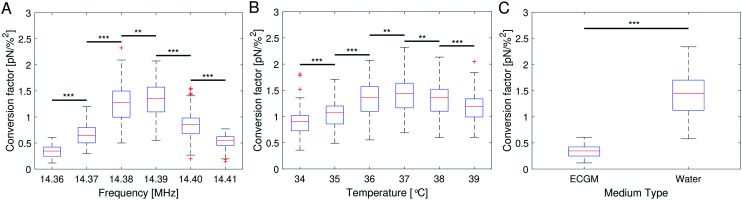
Conversion factors obtained with the SFC with at least 1000 beads (A) for a fixed temperature of 36 °C in ECGM at different frequencies, (B) for a fixed frequency of 14.39 MHz in ECGM at different temperatures and (C) for a fixed temperature of 36 °C, a fixed frequency of 14.36 MHz in water and in ECGM. All measurements were performed in the same field of view. For every condition the conversion factors are randomly split into three subgroups for statistical analysis. The significance stars ** represent *p* ≤ 0.001, ***: *p* ≤ 0.001, using a two-sample *t*-test with three random subgroups per condition after determining a statistically significant difference between the respective condition groups using a one-way ANOVA with *p* < 0.001.

From bottom to top, the AFS chip consists of a bottom glass layer, a fluid layer (approximately 100 μm in height), a top glass layer and a transparent piezoelectric element (piezo) glued on top of the glass layer, see Fig. 1. The glue also serves as a transducer of the acoustic energy to the glass chip. For a fixed chamber geometry design, there is only a given set of usable resonance frequencies.^24^ The resonance frequency at which the first *z*-node, *i.e.* the *z*-position where the force equals zero, is between 10 μm to 20 μm is the frequency of the sinusoidal voltage signal applied to the piezo. The performance of the AFS is therefore sensitive to changes in the layer thicknesses. For our chips the acoustic resonance frequency ranges from 14.30 MHz to 14.40 MHz depending on the individual chips. It is expected that the forces decrease if a frequency different to the resonance frequency is used. This is confirmed by performing the SFC to create a spatial map at the same FoV and a fixed temperature for different frequencies in Δ*f* = 0.01 MHz steps (Fig. 4A). As expected, the conversion factor depends sensitively on the applied frequency.

Eqn (8) suggests that a change in temperature only results in a change of the absolute value of the acoustic force, as compressibility, density of the medium and bead, and even the volume of the bead are temperature-dependent. To check this, the SFC is performed at the same FoV and a fixed frequency for different temperatures (Δ*T* = 1 °C), while correcting the temperature-dependent viscosity by eqn (17). The result shows an optimal temperature (Fig. 4B) for a fixed frequency. This result indicates that for each temperature there is at least one resonance frequency, which was confirmed by changing both frequency and temperature. ESI† Fig. S5† shows that the resonance frequency increases with increasing temperature in the measured temperature range of 25–40 °C.

The speed of sound of a material depends on the compressibility and the density of the material (eqn (18)). Hence, the resonance frequency and thus the force will differ in different media. To check the influence of media composition, for a fixed frequency and fixed temperature the SFC is performed in water and in cell culture medium (endothelial cell growth medium, ECGM) which is used for later experiments. The result clearly shows a difference in the force depending on the medium (Fig. 4C). The median of the conversion factor for water and ECGM appears to vary by a factor of 4.255, when using the same resonance frequency. ESI† Fig. S6A shows that the resonance frequency inside water is down-shifted compared to the one in ECGM (Fig. 4A).

Our improved calibration method Stokes force calibration (SFC) for the AFS shows that the acoustic force inside the AFS is dependent on at least seven parameters that may vary in different experiments. It is dependent on the *z*-position shown by the force profile (Fig. 2B), on the *xy*-lateral position indicated by the spatial force map (Fig. 3D), on the applied resonance frequency (Fig. 4A), on the temperature (Fig. 4B) and on the medium (Fig. 4C). Without further testing, the acoustic force will also depend on the type of beads, *e.g.* polystyrene or silica, as well as their bead sizes. However, these two bead dependencies may only change the absolute value of the exerted force without shifting the resonance frequency.

### Bead immersion into the cell

**Fig. 5:**
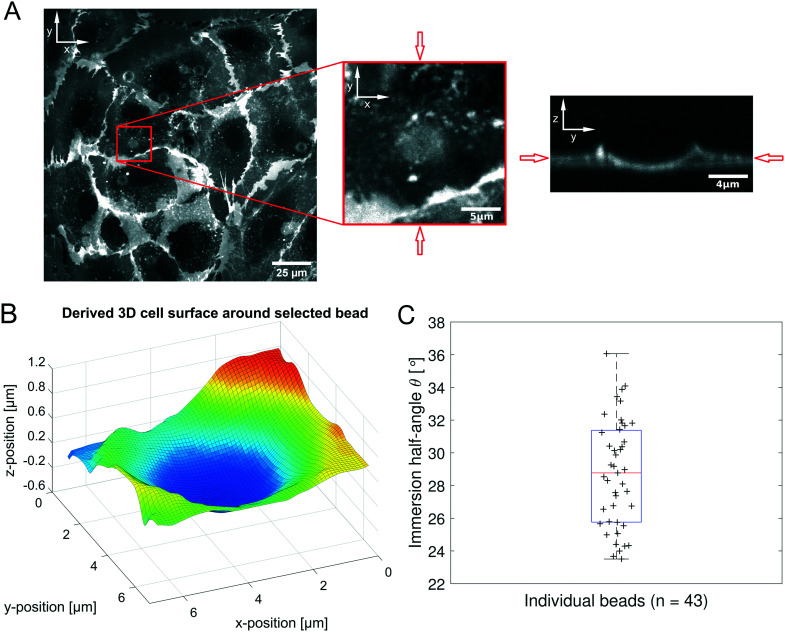
(A): On the left the fluorescence signal of plasma membrane stained HUVECs is shown for the plane of highest intensity of a representative field of view. The middle part highlights a zoomed in selection around an exemplary bead position. On the right the *zy*-slice of the 3D-stack through the bead's center as indicated by the red arrows is displayed. (B): 3D surface plot of the derived cell membrane surface in close perimeter around the center of the bead from (A), color encodes the position along the *Z*-axis for better visibility. (C): Distribution of calculated immersion half-angle *θ* of all measured beads.

While the AFS allows access to both, the force acting on the particle, and the displacement, further information is required to gain a mechanical modulus that can be compared to other measurements and can be modeled by continuum mechanics. To obtain the viscoelastic shear modulus G* the interaction surface between the bead and the cell needs to be determined. This was done by measuring the immersion half-angle from 3D stacks of the fluorescently labeled membrane of HUVECs in a monolayer exposed to attached collagen coated polystyrene beads. Fig. 5A shows the fluorescent signal in the *xy*-plane of highest signal intensity and the *zy*-plane through the bead center for one exemplary bead out of a representative field of view. A 3D-surface plot of the derived cell membrane surface is shown in Fig. 5B. For each bead four different contact points were chosen by eye by estimating the point of turning curvature going outwards from the center of the bead at the deepest indentation. By that, estimating the diameter of the contact circle, the immersion half-angle *θ* was calculated according to eqn (1). Fig. 5C shows the obtained values for every measured bead. The average half-angle was determined to *θ* = 28.73 ± 0.50° (mean ± SEM, *n* = 43).

### Frequency-dependent microrheology using the AFS

A fundamental question in cell mechanics concerns the correct description of cells and tissue with quantitative models. As biological tissue is in general viscoelastic, time-dependent mechanical moduli are expected. Experimentally, such time dependence is best tested in the frequency domain, as the time domain is more susceptible to noise and instrument drift. Up to now, the AFS has not been used for such frequency-dependent measurements, mainly because the software of the manufacturer was not capable of generating the necessary signals. To optimally exploit the potential of the AFS, we wondered if it is possible to perform frequency-dependent measurements by applying several frequencies simultaneously by adding the signals, thus generating a multi-frequency oscillatory (mOsc) force which is applied on the beads with the superimposed frequencies 0.1 Hz, 0.5 Hz and 1.5 Hz. Especially for these low frequencies, the benefits of the superposition of frequencies are noticeable as a sequential application of each low frequency would drastically increase the necessary measurement time. As mentioned in the Materials and methods section, the choice of the frequencies were mainly hardware-related, and can be extended to other frequencies using a faster camera.

To test this new method for microrheology using acoustic forces we oscillate beads attached on a HUVEC monolayer. An important question regarding the viscoelastic properties of HUVEC cells is which model can adequately describe the endothelial cell monolayer. The possibility of culturing a HUVEC monolayer inside the AFS chip with a small volume using our protocol is shown in the ESI† (Fig. S8). It required a constant medium flow which was possible using the microfluidic chip of the AFS.

During the experiment the mOsc force and the medium flow is continuously active. The complex shear modulus G*(*ω*) is calculated using eqn (4). The data was taken from moving time windows of size Δ*t* = 500 s and shift Δ*t*_sh_ = 100 s. The complex shear modulus is calculated at each mOsc frequency. The frequencies chosen represent a common regime used in microrheology, and are easily accessible with the camera based analysis system of the AFS. However, higher or lower frequencies can be used depending on the hardware attached to the AFS.

**Fig. 6:**
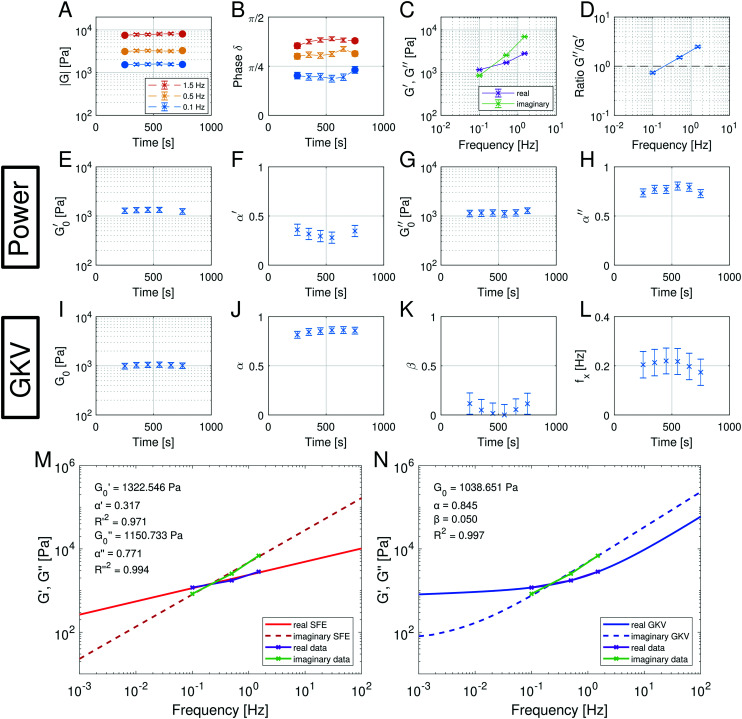
Multi-oscillation microrheology with the SFE model (a power model) and the generalized Kelvin–Voigt (GKV) model. (A and B) Components of the polar form of the complex shear modulus at different time points for three different mOsc frequencies. The filled dots represent independent data results while the crosses represent data with an overlapping time interval. The evaluation time interval is Δ*t* = 500 s and the shift time is Δ*t*_sh_ = 100 s. The average amount of analyzed beads is *n* = 133 in *N* = 9 experiments. Error bars in (A) represent the standard error of the mean and in (B) from error propagation. (C and D) The mean values of the real and imaginary part and their ratio during the force application for different frequencies. Error bars in (C) represent the standard error of the mean and in (D) from error propagation. (E–H) Fit parameters (
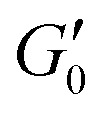
, *α*′, 
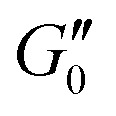
, *α*′′) obtained with the SFE model (power model) which is applied separately for the real and imaginary part of the complex shear modulus. Only fit parameters with a goodness of fit of *R*^2^ > 0.9 are shown. Error bars represent the 2*σ* confidence interval of *n*_boot_ = 100 bootstrap samples. (I–L) Fit parameters (*G*_0_, *α*, *β*) and the calculated crossover frequency *f*_*x*_ = *ω*_*x*_/2π obtained with the GKV model which is applied simultaneously for real and imaginary part of the complex shear modulus. The goodness of fit is *R*^2^ > 0.9. Error bars represent the 2*σ* confidence interval of *n*_boot_ = 100 bootstrap samples. (M and N) Representative data of the real and imaginary part of the complex shear modulus at a time point and fit using (M) the SFE model separately for the moduli and (N) the GKV model simultaneously for the moduli.

Fig. 6 summarizes the results of the viscoelastic properties found for the HUVEC cells using the AFS. The first question we wanted to address was if the viscoelastic properties of a HUVEC monolayer remain constant during the measurement. This is important, as living system often change their properties when exposed to variable force fields. As presented in Fig. 6A and B, we observe that both the absolute value of ∣*G*(*ω*)∣ and the phase angle *δ* vary only marginally over the measurement time. This suggests that the measurements do not disturb the system, and that the AFS can yield reliable and reproducible measurements of viscoelastic moduli. As expected for living cells ∣*G*(*ω*)∣ increases with frequency, and frequency dependency of the phase hints for a different behavior of the real (*G*′(*ω*)) and imaginary (*G*′′(*ω*)) part of the modulus regarding the frequency.

Next, we wondered if the HUVECs follow the power-law rheology that was already reported in many other cell types. Indeed, as demonstrated in Fig. 6C the temporal mean values of *G*′(*ω*) and *G*′′(*ω*) plotted as a function of frequency support the assumption of a power-law in frequency space. Interestingly, we observe a crossover of the storage and loss modulus, suggesting that the mechanical behavior switches from an elastic to a dissipative regime at a frequency of about 0.2 Hz. This is further demonstrated when plotting the ratio of *G*′/*G*′′ (Fig. 6D), that seems to also follow a power-law. In contrast to most currently used models, the logarithmic plot (Fig. 6C) immediately shows that two different power-laws can be seen for *G*′(*ω*) and *G*′′(*ω*) which would correspond to two different power exponents.

The existence of different power-laws suggests that the commonly used model for the viscoelastic biological materials, which is a single fractional element model (SFE) is not consistent with the measurements, as it predicts a single power-law exponent, and cannot explain the found crossover. Nevertheless, we decided to investigate if using a phenomenological approach by applying two independent SFE models each to the storage and loss modulus (*G*′ and *G*′′) would fit the data. Indeed, these phenomenological power-laws fit the data at all the different time points with a high goodness of fit *R*^2^ ≥ 0.9. The power exponents of *G*′(*ω*) are clearly different from *G*′′(*ω*) while the respective values of *G*_0_ are similar.

However, as both the storage and the loss modulus are not independent from each other, but coupled by causality, which is established by the Kramers–Kronig relation,^43^ the resulting phenomenological extension of the SFE model was unsatisfactory, as it describes a non-physical material. Hence, we wondered if a physical extension would provide a better description. We used the generalized Kelvin–Voigt (GKV) model to fit the data simultaneously for both *G*′(*ω*) and *G*′′(*ω*), as this models a two-component material where each component has its individual viscoelastic properties. Indeed, the GKV model even fits the experimental data with higher quality than the phenomenological extension of the SFE model, although it has less fit parameters, see Fig. 6M and N. The fit parameters *G*_0_, *α* and *β* are obtained with eqn (12) and (13) and are shown in Fig. 6I–K at different times throughout the measurement. The mean fit parameter values are found to be *G*_0_ = 1.033 ± 0.132 kPa, *α* = 0.850 ± 0.035 and *β* = 0.058 ± 0.107. The exponent *β* is close to 0, thus at the low frequency limit the shear modulus is independent of the frequency and can be interpreted as a steady state elasticity corresponding to a Young's modulus of *E* = 3.099 ± 0.396 kPa. From the obtained exponents, we can directly deduce the crossover frequency *f*_*x*_ = *ω*_*x*_/2π = 0.204 ± 0.054 Hz with eqn (14) as shown in Fig. 6L. We furthermore tested another fractional model, the generalized Maxwell (GM) model, which was not able to fit the experimental data (ESI† Fig. S11).

These results are the first frequency-dependent measurements on HUVEC monolayers. They allow a stringent test of different, commonly used viscoelastic model descriptions in cell mechanics, and suggest that the GKV model is best describing HUVEC monolayers over a long time. Thanks to the microfluidic design of the AFS, it was furthermore possible to perform these measurements under a constant fluid flow.

### Comparison to optical tweezers

Moreover, we compared the throughput and model results of the AFS and optical tweezers (OT) on HUVECs, see ESI† (Fig. S13). Unlike the AFS, the OT measurements are serial in general, *i.e.* only one bead can be measured at a time. Additionally, the application of each frequency had to be performed sequentially which increased the required measurement time as the superposition of frequencies would require a higher laser power that would then induce higher local heating due to the laser. In our measurements with the AFS, in average we could measure about 15 beads per experiment to obtain values every 100 s, *i.e.* the results correspond to the same time for each bead. Whereas in our measurements with the OT, we could measure one bead in 100 s. Changing the laser and stage position to measure another bead required additional time, in our case several minutes. Suppose these delayed measurements were not relevant, the throughput using the AFS would still be at least 15 times higher than the OT even if one could ideally measure beads sequentially without searching for the next bead or setting up the laser. Atomic force microscopy (AFM) is not expected to be significantly faster. The throughput of a parallelized measurement method like the AFS can even be further increased by increasing the field of view to simply record more beads at the same time. Furthermore, the results of the complex shear modulus obtained with the OT can also be described with the generalized Kelvin–Voigt model as used in this work, see ESI† Fig. S13. As the OT measured phagocytosed beads, it is expected that the found parameters differ from the AFS, which was indeed confirmed in our measurements.

### Dynamical HUVEC stiffness and power-law depend on the actin cytoskeleton

After having established that the AFS is able to perform frequency-dependent measurements of the viscoelastic material properties of HUVEC monolayers using a careful calibration, we wondered if we can also exploit the capacities of the AFS to perform time-dependent measurements of the dynamical changes in cell mechanics upon pharmacological influencing the endothelial cells. An excellent target for this test is the actin cytoskeleton, that can be disrupted by application of cytochalasin B (cyto B). It is known in many other systems that the actin cytoskeleton is a key component for the viscoelastic properties of cells. Thanks to the AFS we can directly monitor both the mechanical changes, and the timescale of these changes in HUVEC during the disruption of the actin cytoskeleton.

**Fig. 7:**
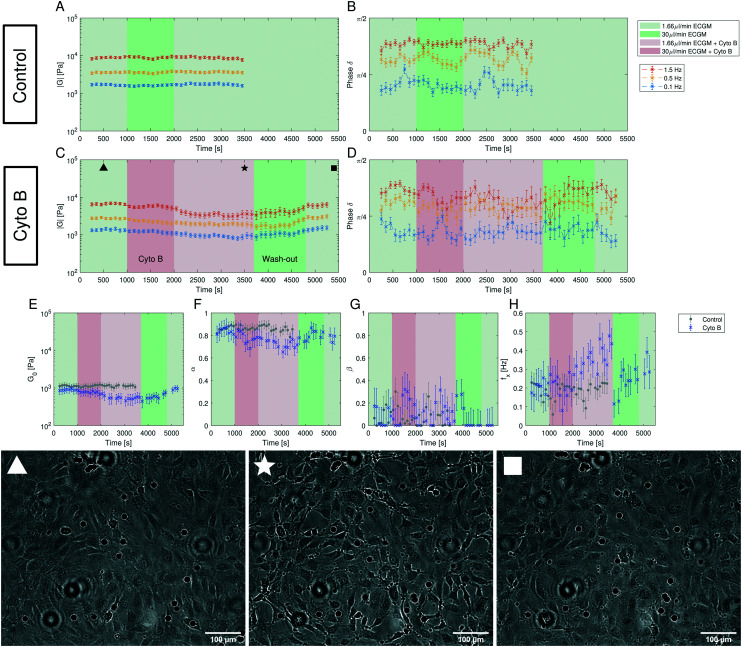
Dynamics of the complex shear modulus measured with multi-oscillation microrheology under flow and cytochalasin B (cyto B) insertion. The marked areas indicate the time intervals of different flow rates with the respective medium composition. All values are shown in Δ*t*_sh_ = 100 s steps at an evaluation time interval of Δ*t* = 500 s. (A and B) The components of the polar form of the complex modulus of a control experiment over 3700 s. For the control experiment only medium (ECGM) has been inserted. The average amount of analyzed beads is *n* = 71 in *N* = 5 experiments. The error bars represent the standard error of the mean. (C and D) The components of the polar form of the complex modulus of the experiment with the addition of cyto B over 5500 s. The cyto B flow has been started at *t* = 1000 s and the procedure to wash it out starts at *t* = 3700 s. The average amount of analyzed beads is *n* = 48 in *N* = 4 experiments. The error bars represent the standard error of the mean. (E–H) The parameters *G*_0_, *α*, *β* and *f*_*x*_ = *ω*_*x*_/2π obtained with the GKV model for both the control experiment (gray) and the cyto B experiment (blue). The mean fit quality is 〈*R*^2^〉 = 0.927, and for all shown fits we have *R*^2^ > 0.7. The error bars represent the 2*σ* confidence interval of *n*_boot_ = 100 bootstrap samples. (▲) Image of the cells with beads during the measurement at the time point 500 s when no cyto B has been added yet, (★) at the time point 3500 s when cyto B has been inserted and the cells change their morphology and lose cell–cell contacts, (■) at the time point 5400 s when cyto B has been washed out and the cells regain cell–cell contacts and form a monolayer again. Images are slightly out of focus for the bead tracking and to increase contrast. Additionally, images have been edited to further increase contrast. Scale bar is 100 μm.

We hence loaded the medium with cyto B while performing the frequency-dependent AFS measurements. The experiments are performed as detailed in the Materials and methods section. Briefly (see Fig. 7), after a 1000 s control measurement we either introduce the treatment (cyto B, red in Fig. 7C) or a control situation (normal medium, green in Fig. 7A), where we flush the new medium into the chip by an increased flow for 1000 s. This is followed by a measurement for 1700 s at a low flow rate where we follow the changes of the viscoelastic properties. If the drug has been applied we perform a wash-out experiment by first flushing medium into the chip with increased flow for 1100 s to ensure no drug is left in the chip, and then measure for 700 s. In Fig. 7A–H the light color represents a low flow rate of 1.66 μl min^−1^ and dark colors represent an increased flow rate of 30 μl min^−1^.

The obtained complex shear modulus is shown in Fig. 7A and B for a control experiment without addition of cyto B and in Fig. 7C and D with the insertion of cyto B. As expected, we see a decrease of the absolute value ∣*G*∣ in the cyto B case that recovers quickly after removing the drug. The effect of cyto B and the recovery can also be seen by the change of the morphology of the cells (Fig. 7) in which upon cyto B insertion the cells lose cell–cell contacts and upon recovery they regain the cell–cell contacts. The change in the complex shear modulus and in the morphology is due to cyto B and not due to the increase in flow rate from flushing in treatment medium as the control measurement also included the increased flow rate. The images of the cells with beads during the control measurement are shown in ESI† Fig. S14, there, the morphological changes as seen upon cyto B treatment is not observed in the control measurement.

Using the GKV model we obtain the parameters *G*_0_, *α*, *β* and *f*_*x*_ = *ω*_*x*_/2π as shown in Fig. 7E–H. The fit quality is typically above *R*^2^ = 0.9. ESI† Fig. S14 shows the measurement color-coding the fit quality which shows a decrease of fit quality during the recovery period (4000 s to 4800 s). As expected, by perturbing the actin cytoskeleton by adding cyto B, the parameter *G*_0_ representing an apparent shear modulus clearly decreases to around 50% of its initial value (Fig. 7E). The recovery can also be seen by the following increase of *G*_0_ after washing out the drug. The morphology of the cells also reverts back to their initial state as they regain cell–cell contacts. Moreover, the crossover frequency *f*_*x*_ appears to increase under the effect of cyto B (Fig. 7H). Interestingly, the power-law exponent *α* decreases during the treatment, while *β* remains rather unaffected (Fig. 7F and G). Therefore, the effect of cyto B may affect the complex modulus at higher frequencies more than in the lower frequencies. In the beginning of the experiment, the control and treatment values remain within the error obtained from bootstrapping.

## 4 Discussion

### Reliable calibration of AFS is essential for microrheology

As we demonstrate, care must be taken during the AFS calibration, where the correct viscosity correction is critical for reliable force measurements. Besides this, possibly the most critical observation we made is that the force distribution inside the AFS chip is highly inhomogeneous (Fig. 3D). Previous approaches to use the AFS as microrheology tool assumed that the force calibration is independent of the position. Our finding of a large variability might also explain why the reported calibration values show a large spread as the force values differ at different locations inside the same field of view. More importantly, the force values and distribution also differ in different fields of view of the same AFS chip, see ESI† Fig. S4. This spread however means, that ignoring the spatial distribution leads to an serious increase in variance of the measurements that is not necessary if using the information about the local calibration values. The argument of measuring a high amount of beads and then calculating the mean of the result typically would not solve this issue, unless using the same and very small FoV in which the calibration factor is constant, see ESI† Fig. S4. However, in this case only a small amount of experiments can be performed in parallel, effectively destroying the advantage of the AFS. The spatial map of conversion factors shows a force distribution that is highly inhomogeneous but not randomly distributed and thus is easy to be corrected in experiments. Misleading and severely wrong results could occur when comparing experiments performed at different positions. Therefore, it is very important to perform experiments in calibrated FoV for correct quantitative results or at least in the same FoV for qualitative conclusions.

### Optimal driving frequency depends on temperature and medium

Besides the correct calibration, we have shown that the resonance frequency strongly affects the applied forces (Fig. 4A). Although the manufacturer provides a recommended frequency, it should only be regarded as a rough estimate because the resonance frequency depends on many parameters of the experiments, like temperature and medium. For a quick calibration of the resonance frequency at the set temperature and medium, it is sufficient to measure a few beads with the SFC (see ESI† Fig. S5B). The spatial map as in Fig. 4 can be established once the optimal frequency has been determined. As we have shown that the acoustic forces inside the AFS strongly depends on several parameters, it is important to calibrate the AFS chip specifically for different sets of conditions, *e.g.* temperature or medium, if the conditions are changed in the experiment. For example, it does not suffice to calibrate at room temperature and then performing experiments at different temperatures using that single calibration.

The temperature inside the flow chamber did not change to a high degree (Δ*T* < 0.1 °C) during the SFC where the amplitudes are low and the forces are only applied for about 1 s, see ESI† Fig. S7A. However, at higher amplitudes the temperature will rapidly (minute timescale) rise inside the flow chamber up to a steady state (ESI† Fig. S7B). As shown, this affects the resonance frequency and thus the forces applied on the bead. Hence, it is important to include knowledge about the temperature during the experiments at high amplitudes. In the current design of the chips the temperature sensor was not located near the flow chamber and thus could not detect changes of the temperature inside the flow chamber. The temperature shown in the LabVIEW software is therefore not the temperature inside the flow chamber, but of the exterior of the chip. During a force application the temperature inside the flow chamber has to be measured with an external sensor prior or during the experiment.

### Effect of temperature dependency of high amplitude experiments

Experiments that require an application of a high amplitude will need to challenge the fact that the forces change during the rise of temperature caused by the applied pressure. This can be solved by applying a long-lasting force so that the temperature reaches a steady state for which the chip has been calibrated. For instance, in our relatively long experiment, the temperature at start of the experiment was set to around 33.5 °C. After around 50 s of force application the steady state was reached and the temperature was around 36 °C, see ESI† Fig. S7B. The force at that temperature was then known and did not further change due to the temperature. However, in many other experiments the forces are not applied for a long time. A solution to this could be to apply an acoustic pressure outside of the resonance frequency to exert very low, or hardly any, forces which only increases the temperature by acoustic pressure. Then during the existing acoustic pressure the frequency is changed to the resonance frequency for the given time to apply the desired force. A calibration to determine whether the change in temperature from the one frequency to the resonance frequency is still high should be conducted for this approach. Another solution would be to obtain the temperature-dependency of the force as shown in Fig. 4B. For this approach the change in temperature during the application of the acoustic force has to be known and several spatial calibration maps have to be measured depending on the change in temperature. However, since our experiments were not falling into these categories, they were not further tested.

### Detailed calibration protocol

To summarize the requirements for the usage of the AFS as a measurement tool for experiments, the AFS chips have to be calibrated specifically for an experiment with a set of conditions. A protocol of the SFC is shown in the ESI.† Typically the choice of medium, bead and temperature are set for the experiment and thus at first, the frequency with a suitable force range has to be found using the SFC. Then, a FoV is chosen which preferably has a high force and a low amount of particles on the piezo that disturbs the tracking. This FoV has to be calibrated by creating the spatial calibration map with the SFC. The temperature change inside the flow chamber by application of the acoustic force has to be measured with an external temperature sensor to ensure that the temperature does not change at a high amount or if it does, then countermeasures as described earlier should be taken. For the analysis of the experiment the lateral position of the bead has to be recorded and compared to the calibration map. The amplitude value sent to the piezo can then be converted to the correct force with the spatial calibration map and possibly a temperature correction.

As previously mentioned, the coupling of the signal to the piezo and the coupling of the piezo to the glass *via* the glue is important for the applied force. Thus, the performance of the AFS chip can degrade over time, typically in a timescale of months, which is accelerated inside a humidified incubator. The degradation may result in a weaker force, a change in the resonance frequency or other aspects affecting the applied force. This means that the chip has to be calibrated frequently depending on the surrounding environment, however, the degradation was not observed during single measurements.

### Novel method enables high-throughput microrheology during flow

Thanks to the improved calibration it is now possible to perform quantitative, long term, frequency-dependent rheology measurements under shear flow with high throughput. Using classical techniques, such as optical tweezers, or AFM, only a single measurement can be performed at a given time. In contrast, using our field of view of 678.40 μm × 542.72 μm we could measure about *n* ≈ 15 beads per experiment (>3700 s) which is more than an order of magnitude higher compared to other methods, such as optical tweezers and atomic force microscopes that typically only would be able to measure one bead per experiment. This is especially important when the dynamic changes of a biological system over a long time is of interest. In this case long term experiments are currently not reported at all, because the throughput of classical techniques is too low. In the case of the AFS, the throughput can be even further increased, for example by increasing the size of the field of view. Thus, especially considering long experiments with changes in the conditions that affects the sample globally, such as the insertion of a drug, the AFS is highly suitable in regards of measuring multiple beads simultaneously.

Besides the higher throughput, using the AFS for microrheology also allows to measure samples under a continuous medium flow due to the microfluidic design. This turns out to be crucial for questions regarding the mechanical properties of HUVECs, or potentially also other endothelial cell types. The possibility of flow application turned out to be important for the generation of the HUVEC monolayer, as in the small volume of the AFS chips (6 μl) nutrients become a limiting factor. We find that a very slow flow of 1.66 μl min^−1^, corresponding to a wall shear stress of 5.9 mPa (eqn (20)) is sufficient to grow a monolayer over several days (see ESI† Fig. S8). Without continuous medium exchange, the cells do not form a monolayer (see ESI† Fig. S9).

An additional point is that the perfusion rate was absent in the force calibration, *i.e.* the situation between calibration and measurement may be different. However, since the speed of sound generating the waves is more than five orders of magnitude faster than the motion of the liquid, the actual pressure gradients are not influenced and the calibration in the static situation is valid for the measurement.

### Frequency-dependent measurements using the AFS quantifies viscoelastic properties of HUVECs

In order to perform the multi-oscillation (mOsc) microrheology beads were inserted and attached to the HUVECs. Only beads that were strongly attached to the cells and did not detach due to flow shear stress nor application of the force were tracked. The effect of different bead coatings might be of interest for further research.

HUVEC cell monolayers are optimal cells for the AFS measurements regarding their flat spreading which leads to a low cell contribution in the bright-field images, hence enabling excellent bead tracking on their surface. Additionally, the height of HUVECs usually does not exceed 3 μm at the cell body and is ≥1 μm at cell periphery,^44^ and thus, the force profile in *z* obtained from the calibration can be regarded as constant in *z*-direction (Fig. 2B). However, due to the motility of the HUVECs the lateral position of the beads also changed during the measurement. This has been accounted for by correction of the calibration factor using the calibrated spatial conversion factor.

During the measurement shown here, the lateral forces in *xy* have been small compared to the *z*-force. However, in case of lateral forces displacing the bead in the *xy*-plane during the measurements, the same adjustment of the force can be performed according to the current position of the bead and the calibrated map. In this situation the calibration needs to also take the lateral movement into account. However, this is rather a simple problem, as one needs to keep track of the *xy*-position which is directly delivered by the analysis, and then perform a realigning step during the generation of the map.

In the frequency-dependent mOsc microrheology measurements we span more than one order of magnitude in a single measurement (0.1 Hz, 0.5 Hz and 1.5 Hz). Using more frequencies is possible but either leads to lower signal per frequency or requires an increase in amplitude which also increases the local heating. Higher frequencies would require a faster camera for tracking.

Up to now, no frequency-dependent microrheology experiments have been performed on HUVEC monolayers. We find a power-law rheology consistent with previous measurements on cells,^20,45^ and the overall elasticity values in the kPa regime comparable to previous measurements using AFM.^19,46^ Favorably, the choice of the frequency values enabled the resolution of a crossover event at which the real and imaginary part of the complex shear modulus are equal (Fig. 6C). Classical models using one single fractional element to model power-law materials fail to explain such crossovers. On the contrary, the generalized Kelvin–Voigt (GKV) model, comprised of two single fractional elements in parallel, was able to provide an excellent fit to the data. Here, the full complex curves are explained by only three parameters. The found values of *G*_0_ can be interpreted as steady state elasticity, as the second power-law exponent *β* is found to be close to 0 and in the long time limit the elastic modulus becomes independent of the frequency (see ESI† Fig. S11). The found elastic shear modulus of *G*_0_ = 1.03 ± 0.13 kPa corresponds to a Young's modulus of *E* = 3.09 ± 0.39 kPa assuming a Poisson ratio of 0.5.

This measurement is in excellent agreement with previous measurements of HUVEC cortical stiffness at cell body using AFM yielding a Young's modulus in the range of 2.70–3.23 kPa.^47–50^

Thanks to the frequency-dependent measurements and the choice of the frequencies (0.1 Hz, 0.5 Hz and 1.5 Hz) we determine a crossover frequency at *f*_*x*_ = (0.204 ± 0.054) Hz which suggests that the mechanical properties at the membrane of HUVECs undergo a solid to liquid transition in the second timescales. This is at a much slower timescale as reported for other cell types where the transition on the cortical level happens in the millisecond regime.^51^ Whether this is relevant for the function of endothelial cells that are exposed to variable shear stress has to be determined in further studies.

### Continuous measurements under flow enables dynamic monitoring of viscoelastic properties

The advantage of the AFS is that it makes use of a microfluidic chip, meaning a closed system that allows a fluid flow. Drugs, such as cytochalasin B (cyto B), can be flushed inside during the measurement, *i.e.* during the application of the force, to capture the dynamics of the complex shear modulus without stopping the measurement. Although the effect of disrupting the actin cytoskeleton on cell mechanics is well established, direct monitoring during the treatment flow and measurements of HUVEC mechanics have not been possible previously. Our results show that long (more than 3700 s) application of the mOsc force did not affect HUVEC mechanics. In principle, the measurement time is not limited to our choice of measurement time as long as the medium supply for the cells is sufficient.

It is reported that shear flow has an effect on cell mechanics at a high shear stress, *e.g.* 2 Pa,^52^ however, in our switch of the low flow rates for the cell culture condition to treatment insertion, the corresponding wall shear stress only increased from 5.9 mPa to 106.1 mPa. The calculation of the shear stress is described in the appendix. These shear stresses arising from our flow rates are intentionally kept low to avoid effects to the cells, as it is shown that a wall shear stress of 320 mPa (ref. 53) for 24 h did not induce cell alignment or change in protein expression. In our case the shear stress is at least three times smaller, thus we expect no effect due to the induced shear forces. Indeed, in our experiment we did not observe changes in the viscoelastic properties during the recording time (see Fig. 7A and B). Thus, our change of the flow rates and the resulting increased wall shear stress do not visibly affect the viscoelastic behavior of the HUVEC. In contrast, upon application of cyto B, the complex shear modulus decreased significantly (Fig. 7C and D) which also could be captured using the GKV model shown in the significant decrease of the apparent shear modulus *G*_0_ to almost 50% of its initial value (Fig. 7E and ESI† Fig. S15A). We demonstrate here that already cytochalasin B shows a measurable effect, although it is among the mild and less potent actin polymerization inhibitors. Hence, we expect that other actin perturbing drugs, such as cytochalasin D, may yield qualitatively similar results and might be of interest for further research.

### The interpretation of the GKV model

Overall, we showed that the mOsc microrheology can be applied using acoustic forces and that the data can be analyzed with the GKV model which yields an apparent shear modulus *G*_0_ and the crossover frequency *f*_*x*_ obtained by the two power exponents (*α*, *β*). The generalized Kelvin–Voigt model assumes two independent viscoelastic materials that react in parallel to an applied force. A simple example that we suggest for the HUVECs is to consider two main materials that are interpenetrating each other. Thanks to the understanding of structural elements of living cells, it is known that the cytoskeleton consists of a polymer network. This polymer network would in our view generate one of the two viscoelastic components. Of course, in reality the cytoskeleton contains several networks, such as the actin cytoskeleton, the microtubule cytoskeleton and the intermediate filament. The low frequency regime, where we observe the power-law exponent *β* ≈ 0, would directly correspond to the expected more elastic behavior of a cytoskeletal network. The second, fundamentally different structural element would be the crowded environment filling the space in-between the cytoskeleton. Due to the molecular crowding, this material should have more fluid-like characteristics, albeit not that of a perfect fluid. The second power-law exponent of *α* ≈ 0.8 is perfectly consistent with this interpretation. Application of a high frequency, hence fast deformation will mainly push the crowded cytoplasm in-between the porous cytoskeleton, accordingly in this regime we rather probe the more liquid-like properties of the cytoplasm. When applying low frequency deformations at low forces, the dominant force is the elastic deformation of the cytoskeleton, as the liquid can easily flow when applying low fluid velocities in the low frequencies. Here the dominant contribution comes from the elastic cytoskeleton.

This interpretation of two, parallel mechanical structures is further validated by the application of cyto B, which decreases the elastic properties measured by *G*_0_. The here proposed interpretation needs further critical testing, however if confirmed, it allows to independently test the properties of the cytoskeleton and of the cytoplasm in a single elegant measurement.

## 5 Conclusions

Overall, we establish a novel method for multi-oscillation microrheology on cell monolayers using acoustic forces with the acoustic force spectroscopy (AFS) which allowed us to determine the frequency-dependent viscoelastic properties of HUVEC monolayers. The data is well explained with a generalized Kelvin–Voigt model using fractional elements. However, measurements with the AFS require a thorough calibration depending on the experiment, mainly due to the force inhomogeneity and the dependencies of the applied force on the applied frequency, temperature and medium. The calibration result of our work is crucial for most researchers using the AFS for their experiments since an incorrect calibration leads to erroneous quantification and mistaken conclusions. Moreover, we have shown that our new method for multi-oscillation microrheology can provide high-throughput measurements which is at least an order of magnitude higher than usual methods, such as optical tweezers or AFMs. A special feature of the AFS method is that is allows measurement under physiological flow conditions. Our method takes the advantage of the capability to measure inside a flow system enabling the first measurement of the dynamics of the complex shear modulus of HUVEC monolayers under flow, and to determine the time evolution of a drug effect on these cells. Hence, we have opened the door for more complex experiments of microrheology under flow that are typically not possible with other techniques like AFM. Future experiments could be to measure the dynamics at different flow stresses, and the time course of such mechanical stresses, as the mechanical response to shear stress of endothelial monolayers is a key element relevant for vascular integrity and immune response.

## Appendix

### Generalized Kelvin–Voigt model

The generalized Kelvin–Voigt model is simplified and shown in eqn (3) by setting *t*_0_ = 1 s that leads to 
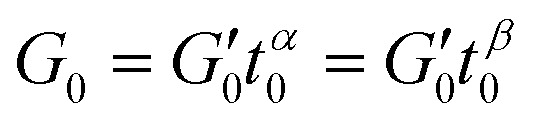
, thus yielding the expression of the real and imaginary part as:12
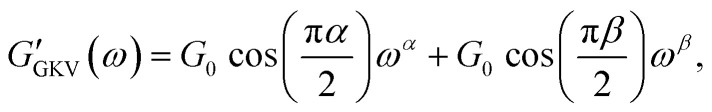
13
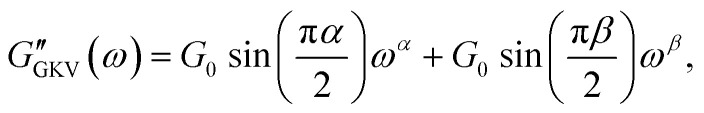
The simplification yields expressions (eqn (12) and (13)) with three independent parameters (*G*_0_, *α*, *β*).

This model gives a crossing of the real and imaginary part at the crossover frequency14
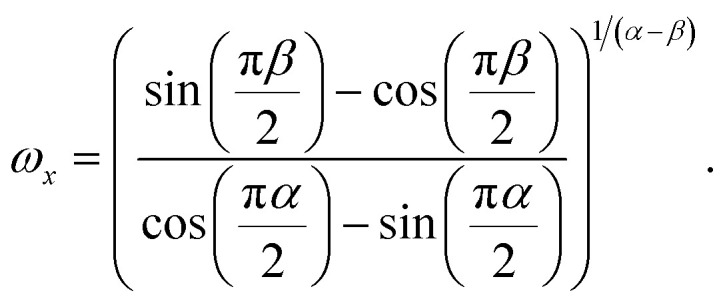


In the ESI† (Fig. S10) certain selected cases for different *α*, *β* are shown for the complex shear modulus obtained with the SFE, GKV and the generalized Maxwell (GM) model.

### Temperature-dependent properties of water

The temperature-dependent density of air-saturated water *ρ*_AS_(*T*) and isothermal compressibility *κ*(*T*) can be approximated with a polynomial of fourth order^54^

15 *f*(*T*) = *A* + *BT* + *CT*^2^ + *DT*^3^ + *ET*^4^

with the constants shown in Table 2 and the temperature *T* ∈ [5, 40] °C. The compressibility-corrected temperature-dependent water density is then given by^54^

16 *ρ*_m_ = *ρ*_AS,T_[1 + *κ*_T_(*P* − 101.325 kPa)]

with the ambient pressure *P* in kPa. The viscosity is also temperature-dependent, *η* = *η*(*T*), and for water it can be approximated with a modified Andrade equation given by^40^17
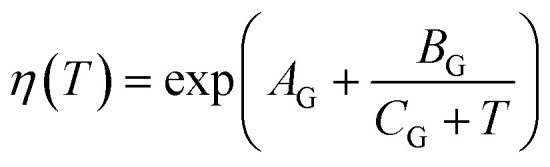
with the constants *A*_G_ = −3.63148, *B*_G_ = 542.05 °C, *C*_G_ = 129.0 °C and the temperature *T* in [°C]. The speed of sound in water can be calculated with the Newton–Laplace equation18
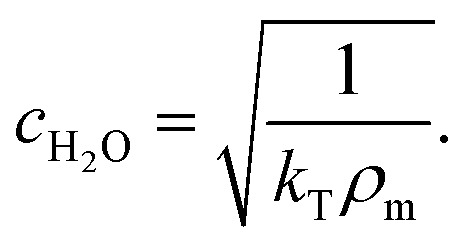


**Table 2:** Values of the constants to calculate the temperature-dependent density and isothermal compressibility^54^

Property	*A*	*B* [1/°C]	*C* [1/°C^2^]	*D* [1/°C^3^]	*E* [1/°C^4^]
Density [kg m^−3^]	999.84847	6.337563 × 10^−2^	−8.523829 × 10^−3^	6.943248 × 10^−5^	−3.821216 × 10^−7^
Compressibility [1/kPa]	50.83101 × 10^−8^	−3.68293 × 10^−9^	7.263725 × 10^−11^	−6.597702 × 10^−13^	2.87767 × 10^−15^

### Viscosity correction due to the surface boundary

The viscosity correction by Faxén's law for translation parallel to the boundary surface is given by^18^19
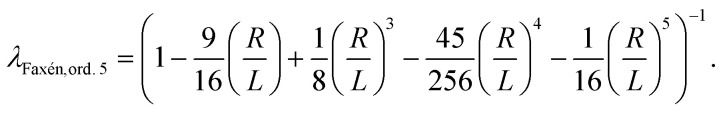


### Wall shear stress from fluid flow

The wall shear stress *τ*_w_ at the center of the microfluidic, rectangular-shaped chamber is estimated by^55^20
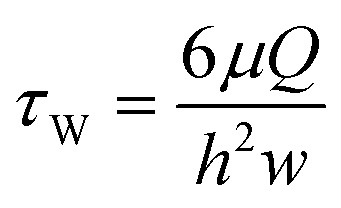
with the viscosity of the medium *μ*, the volumetric flow rate *Q*, the height *h* and width *w* of the chamber.

## Author contributions

T. B. designed the research. M. B. carried out the bead immersion experiment and analyzed its data. T. M. M. and A. N. performed the experiment using the optical tweezers and analyzed its data. A. N. carried out the AFS experiments, analyzed the data, and wrote the original draft. T. B., M. B., T. M. M. and A. N. reviewed and finalized the manuscript.

## Conflicts of interest

There are no conflicts to declare.

## Supplementary Material

LC-021-D0LC01135E-s001

LC-021-D0LC01135E-s002
