# Supplementary material for: Multi-oscillation microrheology via acoustic force spectroscopy enables frequency-dependent measurements on endothelial cells at high-throughput
Source: Lab Chip. 2021 Mar 16;21(10):1929–47. doi: 10.1039/d0lc01135e (PMC8130676; doi:10.1039/d0lc01135e)
Supplement: LC-021-D0LC01135E-s002 [file LC-021-D0LC01135E-s002.pdf]

Electronic Supplementary Material (ESI) for Lab on a Chip.
This journal is © The Royal Society of Chemistry 2021

Supplementary Information

Stokes Force Calibration (SFC)

Theoretical aspects of acoustic radiation force on small particles

The acoustic radiation force F_{rad} on a small spherical particle in a standing acoustic wave in a viscous fluid is the negative gradient of the potential U_{rad} and is given by[2]

$$F_{\text{rad}} = -\nabla U_{\text{rad}}, \tag{S.1}$$

$$U_{\text{rad}} = \frac{4}{3}\pi R^3 \left[f_1 \frac{1}{2}\kappa_m \langle p^2 \rangle - f_2 \frac{3}{4}\rho_m \langle v^2 \rangle \right], \tag{S.2}$$

$$f_1 = 1 - \frac{\kappa_p}{\kappa_m}, \tag{S.3}$$

$$f_2 = \text{Re}\left[\frac{2[1-\gamma(\tilde{\delta})](\frac{\rho_p}{\rho_m}-1)}{2\frac{\rho_p}{\rho_m}+1-3\gamma(\tilde{\delta})} \right], \tag{S.4}$$

$$\gamma(\tilde{\delta}) = -\frac{3}{2}\text{Re}[1+i(1+\tilde{\delta})]\tilde{\delta} \tag{S.5}$$

with the particle radius R, compressibility κ_p and κ_m, density ρ_p and ρ_m of the particle and surrounding medium, respectively. The time average is denoted by $\langle . \rangle$ with the acoustic pressure p and the acoustic velocity v. The dimensionless parameter $\tilde{\delta}$ is given by $\tilde{\delta} = \frac{\delta}{R}$ with the viscous penetration depth $\delta = \sqrt{\frac{2\nu}{\omega}}$ and the kinematic viscosity $\nu = \frac{\eta}{\rho_m}$ given by the ratio of the dynamic viscosity η and the density of the medium, and the angular frequency ω. Similar to the view in[2], for the case in this work the viscous penetration depth is approximately $\delta = 0.15\,\mu$m for a 14.3 MHz sound wave in water at room temperature. The particle radius used in this work is $5\,\mu$m and thus[2] sufficiently large to be described by an inviscid theory where

$$f_2 = \frac{2\left(\frac{\rho_p}{\rho_m}-1\right)}{2\frac{\rho_p}{\rho_m}+1}. \tag{S.6}$$

For a one-dimensional planar standing wave the incoming acoustic pressure and velocity field are given by

$$p(t) = p(z)\cos(\omega t), \tag{S.7}$$

$$\vec{v}(t) = v(z)\sin(\omega t)\vec{e}_z, \tag{S.8}$$

respectively, with the pressure $p(z)$ and velocity $v(z)$ at the position z. With Eq. S.1 and the time averages $\langle \cos^2(\omega t) \rangle = \langle \sin^2(\omega t) \rangle = \frac{1}{2}$ the resulting one-dimensional acoustic radiation force in z is then[3,4]

$$F_{\text{ac}}(z) = -\frac{4}{3}\pi R^3 \nabla \left[\frac{1}{4}\left(1-\frac{\kappa_p}{\kappa_m}\right)\kappa_m |p(z)|^2 - \frac{3}{4}\left(\frac{\frac{\rho_p}{\rho_m}-1}{2\frac{\rho_p}{\rho_m}+1}\right)\rho_m |v(z)|^2 \right]. \tag{S.9}$$

Different viscosity corrections to calculate the acoustic force

An approximation of the viscosity correction by Brenner (Eq. 7) for translation perpendicular to the boundary surface is given by[1]

$$\lambda_{\text{Brenner, ord. 3}} = \left(1 - \frac{9}{8}\left(\frac{R}{L}\right) + \frac{1}{2}\left(\frac{R}{L}\right)^3\right)^{-1}. \tag{S.10}$$

Figure S.1 shows the fits with different viscosity corrections. The validation of the corrections by observing the bead falling and predicting (Fig. S.2A) or fitting (Fig. S.2B) the z-position shows that neither different orders of Faxén's law nor a third order Brenner correction is able to correctly describe the motion of the bead, see Fig. S.2 and Table S.2.

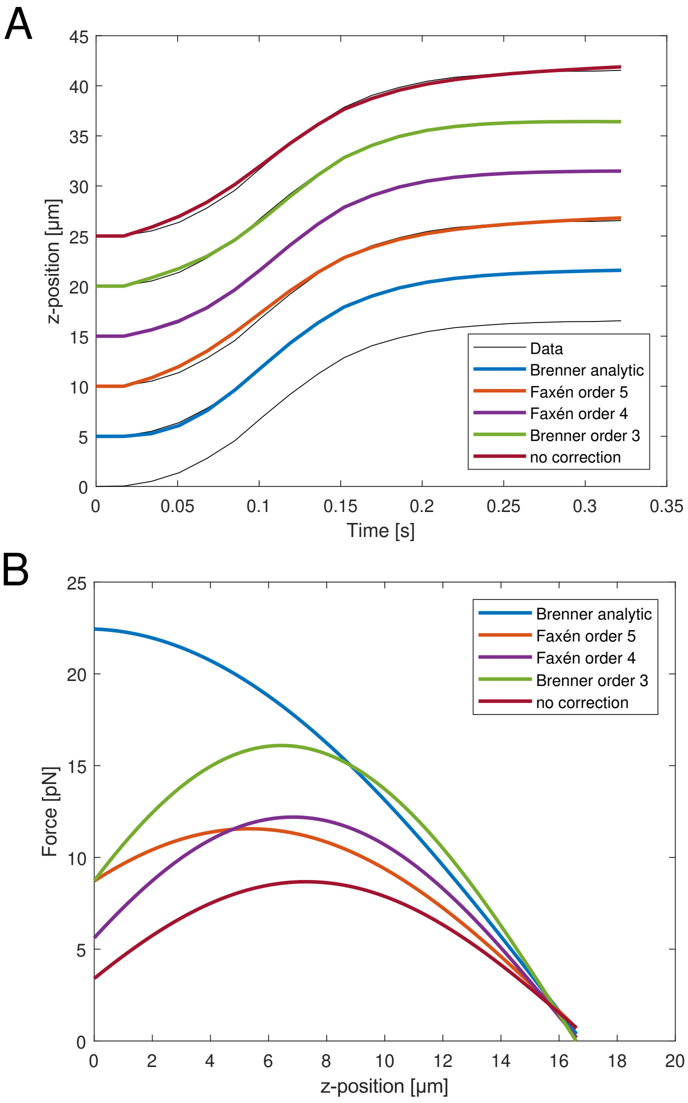

Fig. S.1 (A) Representative, measured z-position data (black line) obtained when turning on the acoustic force during the SFC measurement; the bead moves upward towards the acoustic node. The fits can be obtained by integrating Eq. 10 with the different viscosity corrections. The goodness of fit is $R^2 > 0.9971$ for all shown fits. For clarity, the different fits and the data were shifted by $5\,\mu$m from each other and the first data line only shows the raw data without the fit. (B) Resulting force profile obtained from the fit parameters with Eq. 8 of the positional data from (A) with the different viscosity corrections. The fit parameter values are shown in Table S.2. The applied amplitude was 4%.

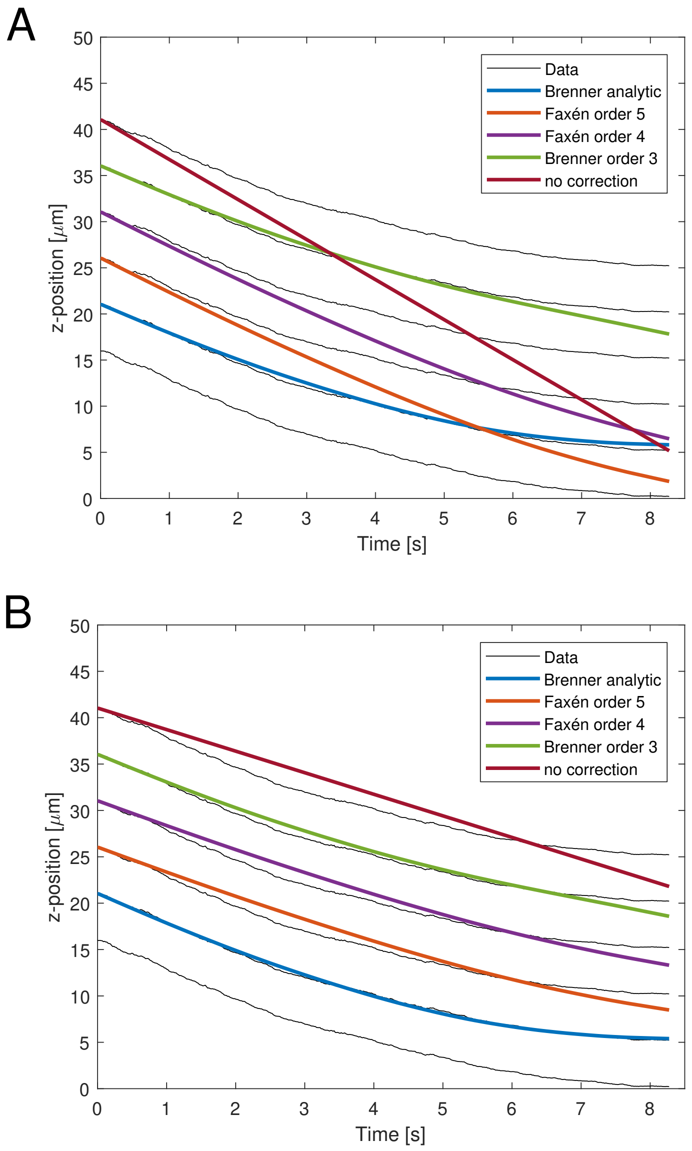

Fig. S.2 Representative, measured z-position data (black line) of a falling bead after turning off the acoustic force. (A) The calculated z-position with different viscosity corrections. (B) The optimized z-position with different viscosity corrections using the density of the bead as a fit parameter. The calculation, fits and data were shifted by $5\,\mu$m from each other for clarity and the raw data is also shown separately. The relevant values are shown in Table S.2.

Lateral translation of free beads

A lateral translation of free beads can be observed when applying an acoustic pressure using the AFS. This can be seen in Supp. Fig. S.3† and in Movie 1†.

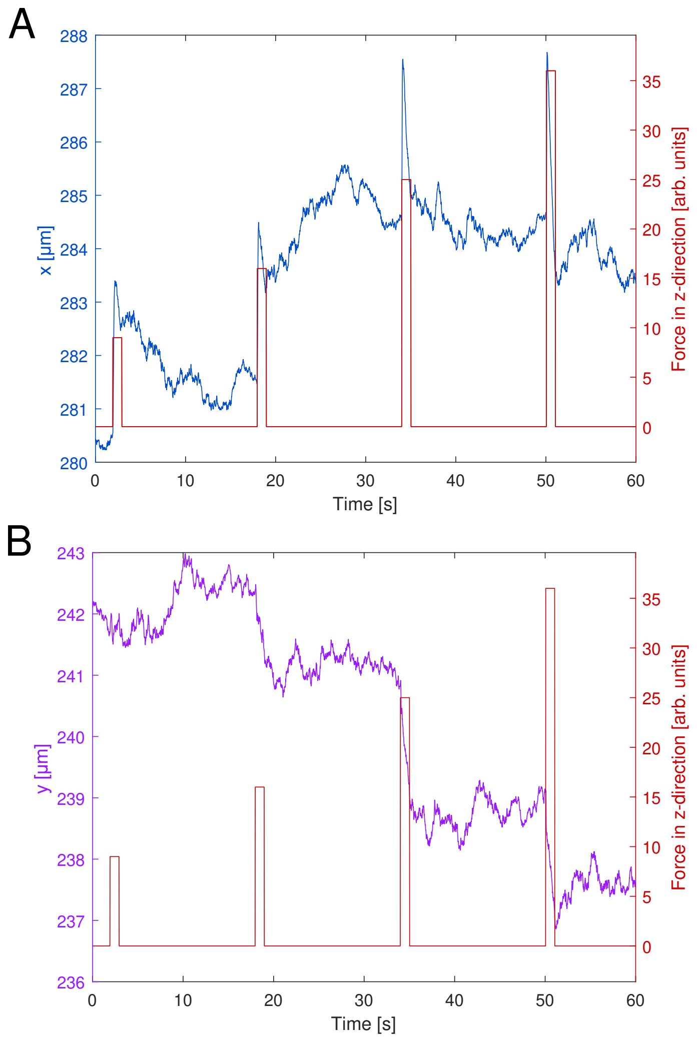

Fig. S.3 The lateral-positional data of the beads during the Stokes Force Calibration. During force application the beads show a lateral translation. The absolute values of the positional data refer to the position of the bead inside the field of view.

Force distribution of different fields of view of the same chip

The force values differ inside a field of view depending on the position which is shown in the spatial calibration map (see Fig. 3D). Additionally, the values even further differ between different fields of view in the same chip as shown in Supp. Fig. S.4†.

Table S.2 Values of the fit parameters to calculate the force profile and the goodness of fit obtained by the SFC and the bead fall shown in Fig. S.1 and Fig. S.2

Correction	f_0 [pNμm]	k_p $\left[\frac{1}{\mu m}\right]$	ϕ_p [rad]	ρ_p $\left[\frac{kg}{m^3}\right]$	R^2_{SFC}	$R^2_{fall, calc.}$	$R^2_{fall, fit}$
Brenner analytic	488.189	0.046	0.799	1051.55	0.9997	0.994	0.998
Faxén order 5	172.300	0.067	0.427	1034.52	0.9971	0.176	0.968
Faxén order 4	152.875	0.080	0.239	1034.28	0.9999	0.139	0.965
Brenner order 3	207.889	0.077	0.287	1047.62	0.9994	0.975	0.985
no correction	108.433	0.080	0.202	1023.85	0.9977	−3.190	0.899

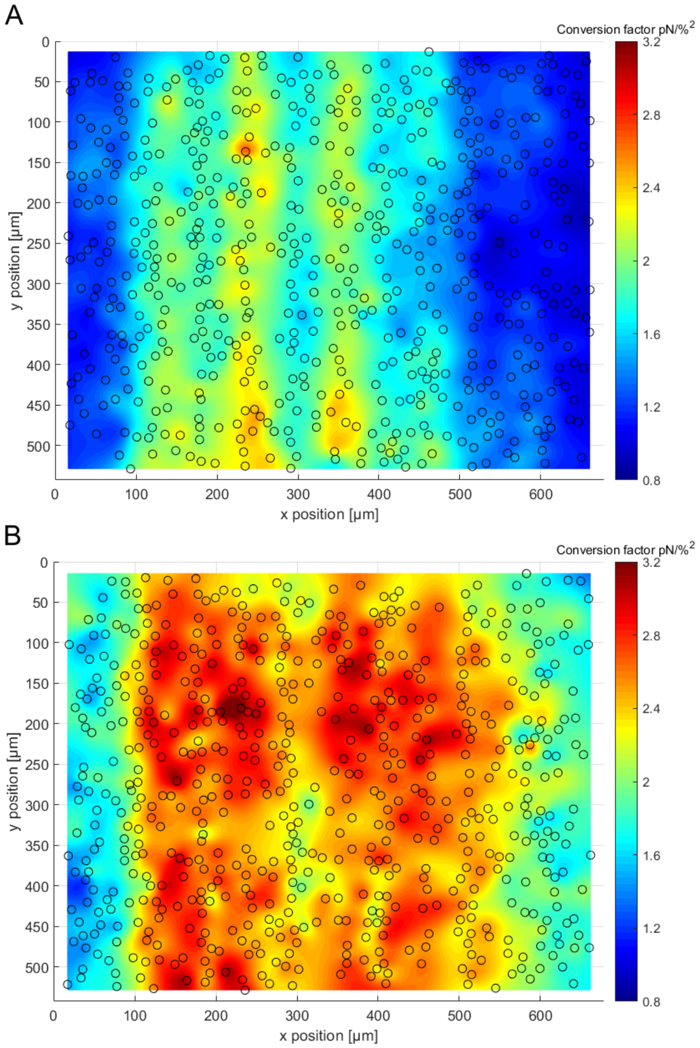

Fig. S.4 Spatial calibration maps obtained by performing the SFC on more than 1000 beads per map at the same conditions and chip at two different fields of view (A and B represent one field of view, respectively). The color scale is the same for both calibration maps.

Data analysis of the SFC

In order to obtain a correct calibration map some beads were filtered out using our software *Kitsune* (available on request); the filter criteria are presented in the following. The raw data obtained with the modified LabVIEW software is an ASCII-file containing the xyz-position, the applied amplitude value $V_{\%}$ and the frequency f at a time point t and a force type value F_t with $F_t = 0$ at time points when no voltage signals were sent to the piezo and $F_t = 7$ for time points when a pulling force calibration and $F_t = 5$ for a multi-oscillation force. For the SFC analysis the time points with $F_t = 7$ were analyzed. The first time point with $F_t \neq 0$ was set to $t = 0$ s. The z-data was shifted by an offset such that $z(t = 0) = 0\,\mu$m. Firstly, the following criteria had to be fulfilled during the applied constant force before being further analyzed.

- There are no z-data points that equal zero which typically indicate tracking errors.
- There are no sudden changes in z, i.e. between each sampling point the difference must be lesser than $10\,\mu$m.
- The maximal z-data must differ by at least $5\,\mu$m above the surface to filter out attached beads or weak forces that could not lift the bead as high during the force application time of 1 s.

The relevant z-positional data is from the ground to the z-node, i.e. the z-position at which the force equals zero. However, the acoustic forces inside the AFS is typically also exerted laterally. Therefore, the bead is also displaced laterally. The force is typically still applied even when the bead has reached the z-node, but due to a possible slight tilt of the flow chamber or a xy-position-dependent z-node the z-position of the bead may gradually change. This is taken into account by only analyzing the start-point up to the time point when the bead first reaches the z-node. For the estimation when the z-node is reached the following steps were performed.

- The full uncropped range is used and Eq. 16 is used to obtain the optimal fit parameters (see description in the Results section).
- The obtained velocity is numerically differentiated to obtain the acceleration.
- The first z-node estimation is the z-position at the time point when the absolute of acceleration is minimal.
- If the first z-node estimation is $< 13\,\mu$m it is set to $13\,\mu$m.
- New optimal fit parameters are now obtained with the z-data from the start point to the first z-node estimation.
- If the fit quality, here, represented by the sum of the squared difference of the numerically obtained z_{num} and the measured z_{meas}, is greater than $1\,\mu m^2$ the z-node estimation is decreased by $0.1\,\mu$m until the fit quality is smaller than $1\,\mu m^2$ or the z-node estimation is smaller than $13\,\mu$m.

With the obtained fit parameters the acoustic force profile can be calculated with Eq. 9. The calibration values are saved to be run through the following filters to create the final calibration map. The values obtained from that amplitude for that bead are filtered out if at least one of the following criteria of *Filter-Type 1* is fulfilled:

- The fit quality (represented by the sum of the squared difference of the numerically obtained z_{num} and the measured z_{meas}) is greater than $2\,\mu\text{m}^2$.
- The fit quality is smaller than $0.001\,\mu\text{m}^2$.
- The x-position or the y-position of the bead is $< 0\,\mu$m.
- The force at $1\,\mu$m changes more than 15% of the maximal force.
- The force at $1\,\mu$m is lesser than 0 pN.
- The force at $1\,\mu$m and the maximal force is greater than 80 pN.

After running through the *Filter-Type 1* the next filter is *Filter-Type 2*. Typically, there are at least three different amplitude values for each bead. *Filter-Type 2* filters out amplitude values that yielded a lower force, albeit being a higher value than the previous amplitude value for one bead. The next final *Filter-Type 3* removes values of all beads that only have one amplitude measured after all the previous filters to ensure the correct quadratic fit for the conversion factor where at least two data points are needed. For the remaining calibration values the conversion factors are obtained for each bead by fitting Eq. 17.

Due to the high amount of beads that were not simultaneously tracked they might overlap. Therefore, the mean values of the conversion factor of the beads that are closer than a merge distance of $10\,\mu$m are used. The map is then created by creating a rectangular grid with mesh size of $0.5\,\mu$m starting from the minimal measured xy-position to the maximal measured xy-position. The grid interpolation is based on a biharmonic spline and is performed by the in-built MATLAB function *griddata*.

Temperature-dependency of the resonance frequency

A short version of the SFC was performed inside a field of view with more than 14 beads at different locations. Only one amplitude was used, therefore the force is represented instead of the conversion factor. The measured data were in $\Delta T = 1\,°$C temperature steps and $\Delta f = 0.01$ MHz frequency steps. The mean force of more than 14 beads at different positions in one field of view is shown in Supp. Fig. S.5† with a cubic interpolation using the in-built MATLAB function *griddata*. Supplementary Figure S.5† shows that the resonance frequency depends on the temperature. Moreover, the resonance frequency increases with higher temperatures.

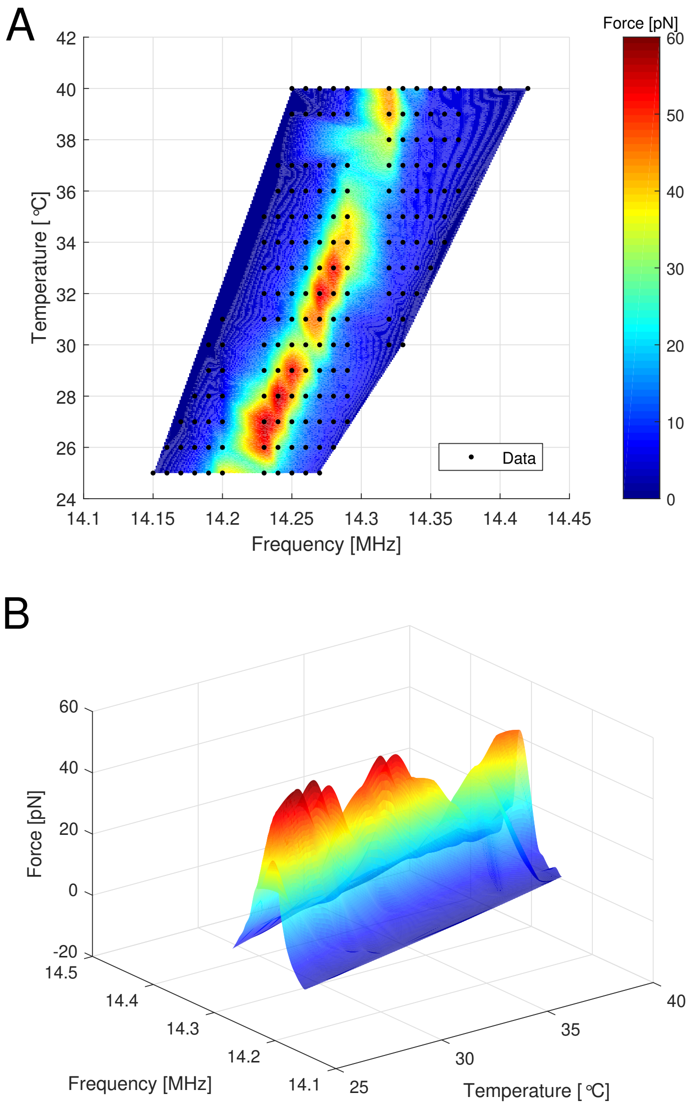

Fig. S.5 Interpolated mean forces on more than 14 beads at different temperatures and different frequencies. (A) 2D-view of the relationship between frequency and temperature. The resonance frequency increases with higher temperature. The black dots present measured data. (B) 3D-view showing the forces at a frequency and temperature.

Resonance frequency shift inside different medium solutions

At a fixed frequency and temperature, the force depends on the medium as shown in Fig. 4C. For a fixed temperature of $T = 36\,°$C the resonance frequency in ECGM was about $f_{\text{res, ECGM}} = 14.39$ MHz for our calibration chip. Supplementary Figure S.6A† shows that the resonance frequency inside water is about $f_{\text{res, water}} = 14.35$ MHz and thus down-shifted compared to the one in ECGM. There is still a difference of the conversion factors in ECGM and in water at a fixed temperature of $T = 36\,°$C at their respective resonance frequencies, however, it is not too high (Supp. Fig. S.6B†) and may be due to the resolution of the exact resonance frequency scan, as it was performed at $\Delta f = 0.01$ MHz steps.

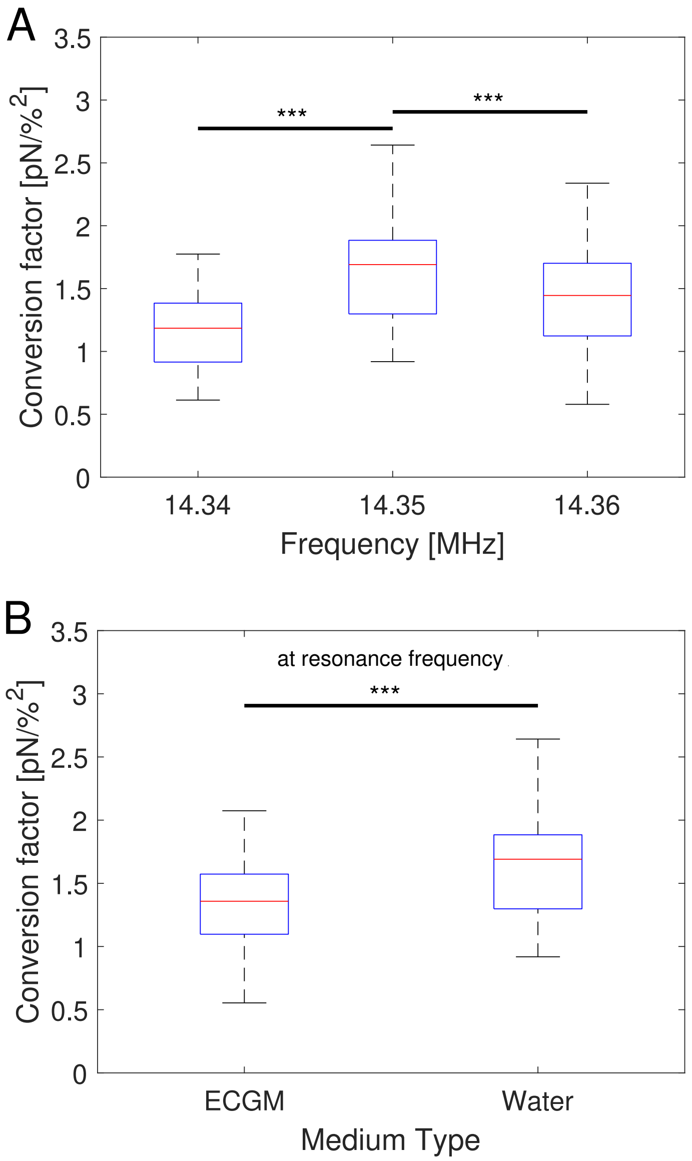

Fig. S.6 Conversion factors obtained with the SFC with at least 1000 beads (A) for a fixed temperature of 36 °C in water at different frequencies, (B) for a fixed temperature of 36 °C in ECGM at the resonance frequency $f = 14.39\,\mathrm{MHz}$ and in water at the resonance frequency $f = 14.35\,\mathrm{MHz}$. For every condition the conversion factors are randomly split into three subgroups for statistical analysis. The significance stars *** represents $p \leq 0.001$ using a two-sample t-test with three random subgroups per condition after determining a statistically significant difference between the respective condition groups using a one-way ANOVA with $p < 0.001$.

Temperature change during force application

It is expected that the temperature changes upon force application. The in-built temperature controller inside the chip holder cannot measure the temperature inside the fluid chamber, but measures the chip's exterior instead. Therefore, an external temperature sensor (disassembled from an AFS G1 chip holder) is used to measure the temperature of the bottom glass underneath the fluid chamber. For this, thermal grease is spread onto the bottom glass. However, care has to be taken of that the thermal grease or the sensor may leave scratches at the bottom of the chip which renders the affected FoVs useless. In addition, if thermal grease is used, the interface glass to air is then changed to glass-grease which typically increases the acoustic energy transmission and thus results in a weaker force. This can lead to a lower temperature increase compared to the situation without the thermal grease.

During the SFC the temperature hardly changes (Supp. Fig. S.7A†). The exact time of the force application during the measurement of 90 s was not known, however, no relevant temperature change could be measured.

When applying higher amplitudes, such as during the mOsc experiment, the temperature drastically increases (Supp. Fig. S.7B†). From around 33.5 °C the mOsc force increased the temperature to a steady state temperature of around 36 °C after about 50 s. Due to the oscillation, the temperature also changed by about $\Delta T = 0.2\,°\mathrm{C}$ which is negligible. The high flow rate of $30\,\frac{\mu\mathrm{l}}{\mathrm{min}}$ of medium at room temperature only slightly decreased the temperature by $\Delta T < 0.5\,°\mathrm{C}$ which is also negligible.

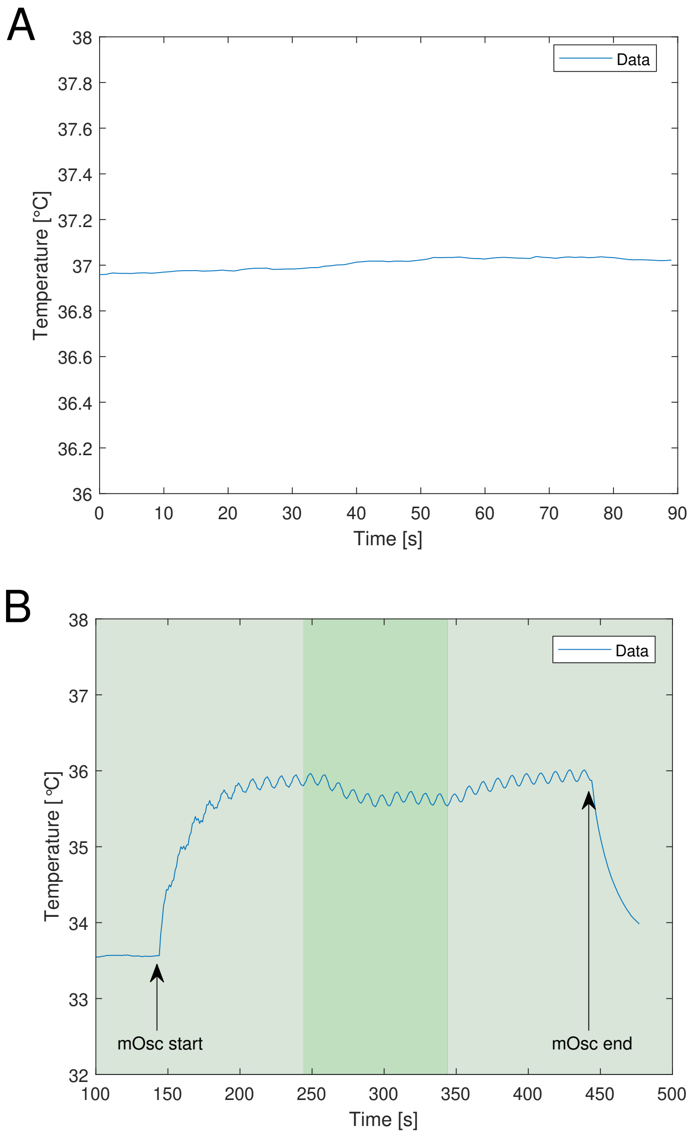

Fig. S.7 Temperature change during force application and flow measured with an external temperature sensor. (A) The temperature during the SFC. The exact time of the SFC forces were unknown, however, inside the force application window (measurement time of 90 s) there is no visible temperature change. (B) The temperature during the mOsc force application. The light green area represents the slow flow of medium ($1.66\,\frac{\mu l}{min}$) and the flow rate in the green area is $30\,\frac{\mu l}{min}$. The mOsc force causes an increase in temperature up to a steady state temperature with a negligible oscillation. The higher flow rate slightly decreased the temperature.

HUVEC culture inside an AFS chip

To demonstrate the possibility of culturing a HUVEC monolayer inside the AFS chip, the cell development is recorded about three minutes after seeding with a sampling time of 1 min. For the recording, the chip is placed on the AFS equipment with the temperature controller instead of the incubator. Due to the closed flow system, the CO_2 environment is not of importance. Figure S.8 shows selected time frames and Movie 2† shows the culture inside the AFS every minute. Inside the AFS chip HUVECs started to spread out onto the surface after about 2 h and were fully spread out a day after seeding. In this case, after 93 h a monolayer of HUVEC had formed. During the incubation, the medium has been renewed with a flow rate of $1.66\,\frac{\mu l}{min}$. The medium renewal was essential to culture HUVEC monolayers inside the AFS (see Supp. Fig. S.9† and Movie 3†). Thus, we show that a monolayer of HUVECs can be cultured inside an AFS chip using our protocol.

Without the renewal of culture medium the cells die before being able to form a monolayer (Fig. S.9†, Movie 3†).

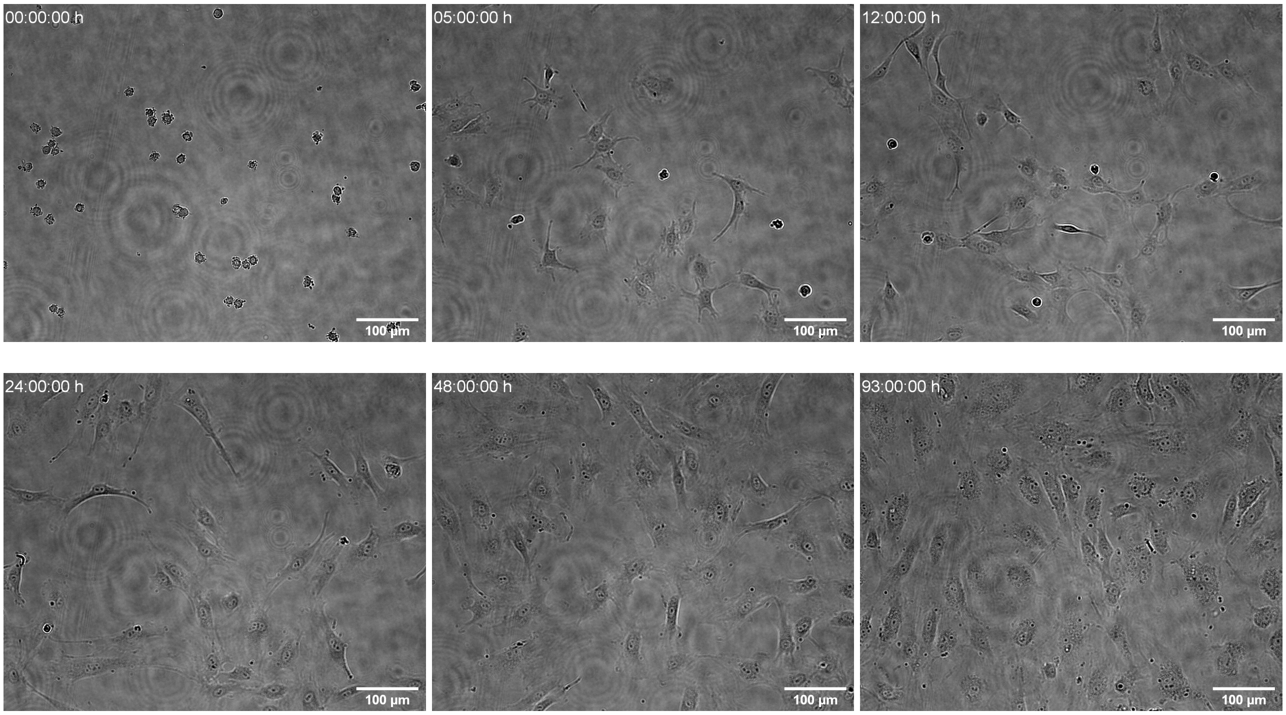

Fig. S.8 HUVEC culture development inside the AFS chip at different time frames. The bright-field images are slightly out of focus to increase the contrast of the cells. Cells are still round three minutes after seeding ($t = 0\,\mathrm{h}$). Cells are fully spread out onto the surface and proliferated ($t = 48\,\mathrm{h}$). A monolayer has formed ($t = 93\,\mathrm{h}$). The images were edited to further increase contrast. Scale bar is $100\,\mu\mathrm{m}$.

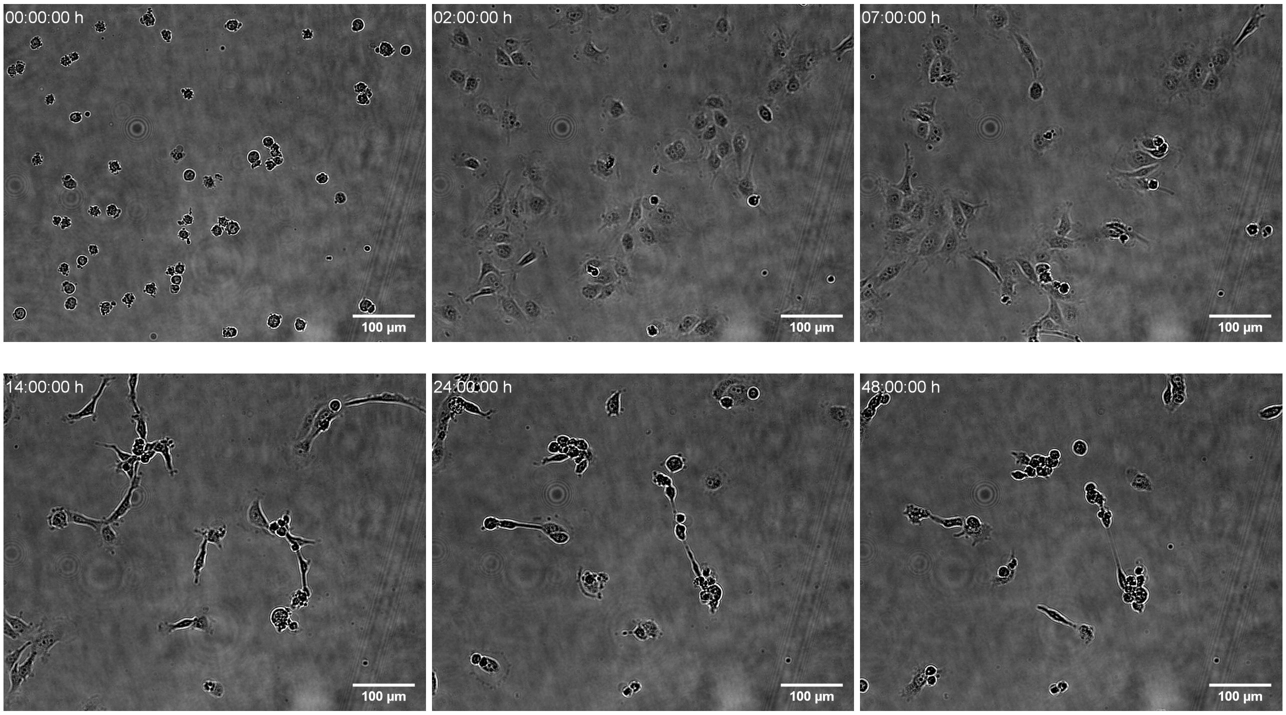

Fig. S.9 HUVEC culture development inside the AFS chip at different time frames without medium renewal. The bright-field images are slightly out of focus to increase the contrast of the cells. Cells are still round three minutes after seeding ($t = 0\,\mathrm{h}$). After $t = 2\,\mathrm{h}$ the cells are spread out onto the surface. After $t = 7\,\mathrm{h}$ the cells start to change their morphology and become elongated. At $t = 14\,\mathrm{h}$ the elongated cells can be clearly seen. After $t = 24\,\mathrm{h}$ the cells round up and die. After $t = 48\,\mathrm{h}$ most of the cells died and the cells are unable to form a monolayer. The images were edited to further increase contrast. Scale bar is $100\,\mu\mathrm{m}$.

Microrheological Experiment

Theory of microrheology

The mechanical properties of complex materials can be measured using microrheological experiments. To interpret the obtained data the microrheological analysis undergoes a few assumptions as follows. The first assumption is that the cell is behaving as a linear viscoelastic material. This can be considered valid for a sufficiently small deformation of the material. For a linear material the superposition principle holds, thus the relation of the strain $\varepsilon(t)$ and the applied stress $\sigma(t)$ has the form of the hereditary integral

$$\varepsilon(t) = \int_{-\infty}^{t} J(t-\tau)\frac{\mathrm{d}\sigma}{\mathrm{d}t}\Big|_{t=\tau}\mathrm{d}\tau, \tag{S.11}$$

with the creep compliance $J(t)$. The Laplace-transformation of Eq. S.11† yields

$$\bar{\varepsilon}(s) = s\bar{J}(s)\bar{\sigma}(s), \tag{S.12}$$

with the complex frequency s in the Laplace-domain, or in the Fourier-domain identify $s = i\omega$, hence

$$\tilde{\varepsilon}(\omega) = i\omega\tilde{J}(\omega)\tilde{\sigma}(\omega). \tag{S.13}$$

Considering an oscillatory applied stress $\sigma(\omega) = \sigma_0 e^{i\omega t}$ of frequency $\omega = 2\pi f$ the resulting steady state strain is also oscillatory $\varepsilon(\omega) = \varepsilon_0 e^{i(\omega t-\varphi)}$ with the same frequency ω, but with an additional phase shift φ. For a purely linear elastic material represented by a single spring element, the phase shift equals zero ($\varphi = 0\,°$) and the strain immediately follows the applied stress; for a purely linear viscous material, a single dash-pot element, the strain lags behind the stress by $\varphi = 90\,°$. Here, the material of interest is viscoelastic and the phase shift is therefore $\varphi \in (0\,°, 90\,°)$. The viscoelastic complex modulus G^* can be defined as the ratio of the stress to strain

$$G^*(\omega) = \frac{\sigma(\omega)}{\varepsilon(\omega)} = \frac{\sigma_0}{\varepsilon_0}e^{i\varphi}. \tag{S.14}$$

The relation between the viscoelastic complex modulus and the creep compliance with Eq. S.13 is then

$$G^*(\omega) = \frac{1}{i\omega\tilde{J}(\omega)}. \tag{S.15}$$

The second assumption is the choice of the model that describes the viscoelastic material. In this case, a model based on fractional calculus is chosen. The following calculations are mostly based on[5]. The underlying assumed element is that the creep compliance increases as

$$J(t) = \frac{J_0}{\Gamma(1+\alpha)}\left(\frac{t}{t_0}\right)^{\alpha} \tag{S.16}$$

with an arbitrary time constant t_0, a constant J_0 and the complete Gamma-function $\Gamma(.)$ for $\alpha \in [0,1]$. With Eq. S.11† and Eq. S.16† the strain is given by

$$\varepsilon(t) = \frac{J_0}{t_0^{\alpha}}\frac{1}{\Gamma(1+\alpha)}\int_{-\infty}^{t}(t-\tau)^{\alpha}\frac{\mathrm{d}\sigma}{\mathrm{d}t}\Big|_{t=\tau}\mathrm{d}\tau. \tag{S.17}$$

With $\alpha = \gamma - 1$ identify the special form of the Riemann-Liouville integral, also known as the Weyl's fractional integral ${}_{-\infty}D_t^{\gamma} f(t) = \frac{1}{\Gamma(\gamma)}\int_{-\infty}^{t}(t-\tau)^{\gamma-1}f(t)\Big|_{t=\tau}\mathrm{d}\tau$, so that

$$\varepsilon(t) = \frac{J_0}{t_0^{\gamma-1}}\,{}_{-\infty}D_t^{\gamma}\frac{\mathrm{d}\sigma(t)}{\mathrm{d}t}. \tag{S.18}$$

The fractional integration and differentiation can be obtained, shown by performing fractional integration followed by ordinary integer differentiation, see Schiessel *et al.*[5], and Weyl's fractional integral can be notated as ${}_{-\infty}D_t^{\gamma} \equiv \frac{\mathrm{d}^{-\gamma}}{\mathrm{d}t^{-\gamma}}$. Re-substituting $\gamma = \alpha + 1$ yields the rheological constitutive equation (RCE)

$$\varepsilon(t) = \frac{J_0}{t_0^{\alpha}}\frac{\mathrm{d}^{-\alpha}\sigma(t)}{\mathrm{d}t^{-\alpha}},$$

$$\sigma(t) = G_0' t^{\alpha}\frac{\mathrm{d}^{\alpha}\varepsilon(t)}{\mathrm{d}t^{\alpha}} \tag{S.19}$$

using the fundamental relations $\frac{\mathrm{d}^{\alpha}}{\mathrm{d}t^{\alpha}}\frac{\mathrm{d}^{\beta}}{\mathrm{d}t^{\beta}} = \frac{\mathrm{d}^{\alpha+\beta}}{\mathrm{d}t^{\alpha+\beta}}$ and $J_0 = \frac{1}{G_0'}$. As coined by Schiessel *et al.*[5] the RCE represents the single fractional element (SFE). The complex modulus can be obtained by Fourier transforming Eq. S.19†

$$G^*_{\mathrm{SFE}}(\omega) = G_0'(i\omega t_0)^{\alpha} \tag{S.20}$$

and with $i^{\alpha} = e^{\frac{i\pi\alpha}{2}}$ and comparing with Eq. S.14 the previously described phase shift is

$$\varphi = \frac{\pi\alpha}{2}. \tag{S.21}$$

Thus, for a purely linear elastic material the exponent α equals zero, for a purely viscous material, $\alpha = 1$, and for a viscoelastic material $\alpha \in (0,1)$. Due to the scaling invariance of Eq. S.20† the time constant is set to $t_0 = 1\,\mathrm{s}$ and $G_0 \equiv G_0' t_0^{\alpha}$. Note that the RCE and the resulting complex shear modulus for the SFE (Eqs. S.19†, S.20†) represent a power-law behavior as often[6,7] used to describe linear viscoelastic materials. The complex modulus can be represented in real and imaginary parts ($G^*_{\mathrm{SFE}}(\omega) = G'_{\mathrm{SFE}}(\omega) + iG''_{\mathrm{SFE}}(\omega)$) or in polar form with the absolute and phase ($G^*_{\mathrm{SFE}}(\omega) = |G^*_{\mathrm{SFE}}(\omega)|e^{i\delta_{\mathrm{SFE}}}$):

$$G'_{\mathrm{SFE}}(\omega) = G_0\cos\left(\frac{\pi\alpha}{2}\right)\omega^{\alpha}, \tag{S.22}$$

$$G''_{\mathrm{SFE}}(\omega) = G_0\sin\left(\frac{\pi\alpha}{2}\right)\omega^{\alpha}, \tag{S.23}$$

$$|G^*_{\mathrm{SFE}}(\omega)| = |G_0|\omega^{\alpha}, \tag{S.24}$$

$$\delta_{\mathrm{SFE}} = \mathrm{atan2}(G''_{\mathrm{SFE}}(\omega), G'_{\mathrm{SFE}}(\omega)), \tag{S.25}$$

whereas the real part represents the elastic storage modulus and the imaginary part the viscous loss modulus. It is noted that G_0 can be regarded as an apparent shear modulus for the time scale of $t_0 = 1\,\mathrm{s}$.

Similar to known mechanical models, such as the Maxwell or the Kelvin-Voigt model, in which springs and dash-pots are arranged in series or parallel, the same arrangements can be applied using single fractional elements instead of simple elements. These arrangements guarantee that the equations provide mechanical and thermodynamical stability and thus are physically meaning-

ful.[5] Two fractional elements can be arranged in series (Generalized Maxwell model, GM) or in parallel (Generalized Kelvin-Voigt model, GKV). The calculations for the GM is shown later in the Supplemental Information (Eq. S.36†). For the GKV, the elements are arranged in parallel, meaning the stresses add and the RCE and the expression of the complex shear modulus for the GKV is

$$\sigma(t) = \left(G_{01} t_{01}^{\alpha} \frac{\mathrm{d}^{\alpha}}{\mathrm{d}t^{\alpha}} + G_{02} t_{02}^{\beta} \frac{\mathrm{d}^{\beta}}{\mathrm{d}t^{\beta}} \right) \varepsilon(t), \tag{S.26}$$

$$G^{*}_{\mathrm{GKV}}(\omega) = G_0'((i\omega t_0)^{\alpha} + (i\omega t_0)^{\beta}) \tag{S.27}$$

with $t_0 = \left(\frac{G_{01} t_{01}^{\alpha}}{G_{02} t_{02}^{\beta}} \right)^{\frac{1}{\alpha-\beta}}$, $G_0' = G_{01} \left(\frac{t_{01}}{t_0} \right)^{\alpha}$ and G_{0i}, t_{0i}, α and β the parameters of the two fractional elements. To simplify the model and reduce the set of parameters, G_0 is regarded to be independent of α and β and $t_0 = 1\,\mathrm{s}$, and since the moduli simply add, the expression of the real and imaginary part is

$$G'_{\mathrm{GKV}}(\omega) = G_0 \cos\left(\frac{\pi\alpha}{2}\right) \omega^{\alpha} + G_0 \cos\left(\frac{\pi\beta}{2}\right) \omega^{\beta}, \tag{S.28}$$

$$G''_{\mathrm{GKV}}(\omega) = G_0 \sin\left(\frac{\pi\alpha}{2}\right) \omega^{\alpha} + G_0 \sin\left(\frac{\pi\beta}{2}\right) \omega^{\beta}. \tag{S.29}$$

The simplification yields expressions (Eqs. S.28†, S.29†) with three independent parameters (G_0, α, β). While for the SFE (Eq. S.20†) there is only one power exponent α, the GKV shows two power exponents (α, β). Therefore, the GKV is able to model crossover events in which at a certain and only frequency the real part of the complex modulus equals the imaginary part. This crossover frequency for a set $t_0 = 1\,\mathrm{s}$ for the GKV is

$$\omega_x = \left(\frac{\sin\left(\frac{\pi\beta}{2}\right) - \cos\left(\frac{\pi\beta}{2}\right)}{\cos\left(\frac{\pi\alpha}{2}\right) - \sin\left(\frac{\pi\alpha}{2}\right)} \right)^{1/(\alpha-\beta)} \tag{S.30}$$

for $\alpha > \beta$ without loss of generality since α and β are interchangeable and $\alpha \in (0.5, 1)$ and $\beta \in (0, 0.5)$. For the special case where $\alpha + \beta = 1$ the crossover frequency is $\omega_x = \frac{1}{t_0}$ ($1\,\frac{\mathrm{rad}}{\mathrm{s}}$ for $t_0 = 1\,\mathrm{s}$). Later, in the ESI† (Fig. S.10†) certain selected cases for different α, β are shown for the complex shear modulus obtained with the SFE, GM and GKV. Briefly, for frequencies $\omega \ll \omega_x$ the complex modulus behaves similar to ω^{β} for $\beta > 0$ and for frequencies $\omega \gg \omega_x$, $G \sim \omega^{\alpha}$. The special case of $\beta = 0$ shows a plateau in the real part of the complex modulus for frequencies $\omega \ll \omega_x$ while the imaginary part is $\sim \omega^{\alpha}\ \ \forall \omega$.

The interpretation of the GKV parameters (G_0, α, β) are the following. G_0 can be considered as an apparent shear modulus at the time scale $t_0 = 1\,\mathrm{s}$ similar to the interpretation of G_0 for the SFE. However, note that $G_{\mathrm{GKV},0} = 2 \cdot G_{\mathrm{SFE},0}$ and is to be considered in a comparison. The power parameters α and β define the crossover frequency ω_x for a set t_0 (see Eq. S.30†). Therefore, the single information of one power parameter merely shows the complex shear modulus of the material over a certain frequency range, while the tuple (α, β) yields information of the complex shear modulus over the whole frequency range for a material with one crossover event.

The model using single fractional elements can be further extended by arranging multiple SFEs, such as the known mechanical models Zener model or Poynting-Thomson model with simple elements. However, this increases the amount of parameters and thus, it will not be used in this framework.

The third assumption for the microrheological analysis is dependent on the experiment. For experiments where the force is transmitted to the sample by an external bead neither the stress σ nor the strain ε are often directly accessible, but the force F acting on the bead and the bead displacement z instead. In the paper by Kollmannsberger *et al.*[6], for a magnetic tweezers experiment, the strain is estimated as the bead displacement divided by the bead radius R and the stress is estimated as the applied force divided by the bead cross section, i.e. the complex modulus $\tilde{G}(\omega) = \frac{\tilde{F}(\omega)}{\tilde{z}(\omega)} \frac{1}{\pi R}$. While in the paper by Balland *et al.*[7], for an optical tweezers experiment, the resulting complex modulus is calculated depending on the immersion of a bead inside the material characterized by an immersion half-angle θ, i.e.

$$\tilde{G}(\omega) = \frac{\tilde{F}(\omega)}{\tilde{z}(\omega)} \frac{1}{6\pi R f(\theta)}, \tag{S.31}$$

$$f(\theta) = \left(\frac{9}{4\sin(\theta)} + \frac{3\cos(\theta)}{2\sin^3(\theta)} \right)^{-1} \tag{S.32}$$

with $[f(\theta) < 1,\ \ \forall\ \ \theta \in (10°, 90°)]$, see Supp. Fig. S.12†, which will be used for the analysis. For a bead immersed in an infinite medium[7,8], the complex modulus would be $\tilde{G}(\omega) = \frac{\tilde{F}(\omega)}{\tilde{z}(\omega)} \frac{1}{6\pi R}$.

Generalized Maxwell Model

As mentioned, the underlying assumption is that the creep compliance behaves as a power law (Eq. S.16†). In the following the results and calculations mostly based on[5] are summarized and presented. For the calculations of the single fractional element (SFE) and the Generalized Kelvin-Voigt (GKV) model, see the Materials and Methods section or the section on the theory of microrheology in the ESI†.

For the Generalized Maxwell (GM) model the fractional elements are arranged in series, meaning the individual strains add, i.e.

$$\varepsilon(t) = \left(\frac{1}{G_{01} t_{01}^{\alpha}} \frac{\mathrm{d}^{-\alpha}}{\mathrm{d}t^{-\alpha}} + \frac{1}{G_{02} t_{02}^{\beta}} \frac{\mathrm{d}^{-\beta}}{\mathrm{d}t^{-\beta}} \right) \sigma(t) \tag{S.33}$$

or with the fundamental relations $\frac{\mathrm{d}^{\alpha}}{\mathrm{d}t^{\alpha}} \frac{\mathrm{d}^{\beta}}{\mathrm{d}t^{\beta}} = \frac{\mathrm{d}^{\alpha+\beta}}{\mathrm{d}t^{\alpha+\beta}}$

$$\sigma(t) = G_{01} t_{01}^{\alpha} \frac{\mathrm{d}^{\alpha}}{\mathrm{d}t^{\alpha}} \varepsilon(t) - \frac{G_{01} t_{01}^{\alpha}}{G_{02} t_{02}^{\beta}} \frac{\mathrm{d}^{\alpha-\beta}}{\mathrm{d}t^{\alpha-\beta}} \sigma(t). \tag{S.34}$$

The Fourier transform yields

$$\tilde{\sigma}(\omega) = G_{01} t_{01}^{\alpha} (i\omega)^{\alpha} \tilde{\varepsilon}(\omega) - \frac{G_{01} t_{01}^{\alpha}}{G_{02} t_{02}^{\beta}} (i\omega)^{\alpha-\beta} \tilde{\sigma}(\omega) \tag{S.35}$$

with the imaginary unit $i^2 = -1$. With $t_0 = \left(\frac{G_{01} t_{01}^{\alpha}}{G_{02} t_{02}^{\beta}} \right)^{\frac{1}{\alpha-\beta}}$, $G_0 = G_{01} \left(\frac{t_{01}}{t_0} \right)^{\alpha}$ the complex shear modulus $G^{*}(\omega) = \frac{\sigma(\omega)}{\varepsilon(\omega)}$ for the GM

model is

$$G^*_{\text{GM}}(\omega) = \frac{G_0 (i\omega t_0)^\alpha}{1+(i\omega t_0)^{\alpha-\beta}}. \quad \text{(S.36)}$$

Microrheological models using fractional calculus

As a compilation, the following equations show the complex shear modulus $G^*(\omega)$, including their real ($G'(\omega)$) and imaginary ($G''(\omega)$) part, in the Fourier domain and the creep compliance $J(t)$ in time domain with $J_0 = \frac{1}{G_0}$ obtained from[5] for the SFE, GM and GKV. Additionally, for the GM and GKV the crossover frequency ω_x for $\alpha \in (0.5, 1)$ and $\beta \in (0, 0.5)$ is shown.
Single fractional element (SFE):

$$G^*_{\text{SFE}}(\omega) = G_0 t_0^\alpha \omega^\alpha e^{i\frac{\pi\alpha}{2}} \quad \text{(S.37)}$$

$$G'_{\text{SFE}}(\omega) = G_0 t_0^\alpha \omega^\alpha \cos\left(\frac{\pi\alpha}{2}\right) \quad \text{(S.38)}$$

$$G''_{\text{SFE}}(\omega) = G_0 t_0^\alpha \omega^\alpha \sin\left(\frac{\pi\alpha}{2}\right) \quad \text{(S.39)}$$

$$J_{\text{SFE}}(t) = \frac{J_0}{\Gamma(1+\alpha)}\left(\frac{t}{t_0}\right)^\alpha \quad \text{(S.40)}$$

Generalized Maxwell model (GM):

$$G^*_{\text{GM}}(\omega) = \frac{G_0 (i\omega t_0)^\alpha}{1+(i\omega t_0)^{\alpha-\beta}} \quad \text{(S.41)}$$

$$G'_{\text{GM}}(\omega) = G_0 t_0^\alpha \omega^\alpha \frac{\cos\left(\frac{\pi\alpha}{2}\right) + \omega^{\alpha-\beta} t_0^{\alpha-\beta}\cos\left(\frac{\pi\beta}{2}\right)}{1+2\omega^{\alpha-\beta} t_0^{\alpha-\beta}\cos\left(\frac{\pi(\alpha-\beta)}{2}\right) + \omega^{2(\alpha-\beta)} t_0^{2(\alpha-\beta)}} \quad \text{(S.42)}$$

$$G''_{\text{GM}}(\omega) = G_0 t_0^\alpha \omega^\alpha \frac{\sin\left(\frac{\pi\alpha}{2}\right) + \omega^{\alpha-\beta} t_0^{\alpha-\beta}\sin\left(\frac{\pi\beta}{2}\right)}{1+2\omega^{\alpha-\beta} t_0^{\alpha-\beta}\cos\left(\frac{\pi(\alpha-\beta)}{2}\right) + \omega^{2(\alpha-\beta)} t_0^{2(\alpha-\beta)}} \quad \text{(S.43)}$$

$$J_{\text{GM}}(t) = \frac{J_0}{\Gamma(1+\alpha)}\left(\frac{t}{t_0}\right)^\alpha + \frac{J_0}{\Gamma(1+\beta)}\left(\frac{t}{t_0}\right)^\beta \quad \text{(S.44)}$$

$$\omega_{\text{GM},x} \overset{t_0=1\,\text{s}}{=} \left(\frac{\sin\left(\frac{\pi\alpha}{2}\right)-\cos\left(\frac{\pi\alpha}{2}\right)}{\cos\left(\frac{\pi\beta}{2}\right)-\sin\left(\frac{\pi\beta}{2}\right)}\right)^{\frac{1}{\alpha-\beta}} \quad \text{(S.45)}$$

Generalized Kelvin-Voigt model (GKV):

$$G^*_{\text{GKV}}(\omega) = G_0 t_0^\alpha \omega^\alpha e^{i\frac{\pi\alpha}{2}} + G_0 t_0^\beta \omega^\beta e^{i\frac{\pi\beta}{2}} \quad \text{(S.46)}$$

$$G'_{\text{GKV}}(\omega) = G_0 t_0^\alpha \omega^\alpha \cos\left(\frac{\pi\alpha}{2}\right) + G_0 t_0^\beta \omega^\beta \cos\left(\frac{\pi\beta}{2}\right) \quad \text{(S.47)}$$

$$G''_{\text{GKV}}(\omega) = G_0 t_0^\alpha \omega^\alpha \sin\left(\frac{\pi\alpha}{2}\right) + G_0 t_0^\beta \omega^\beta \sin\left(\frac{\pi\beta}{2}\right) \quad \text{(S.48)}$$

$$J_{\text{GKV}}(t) = J_0 \left(\frac{t}{t_0}\right)^\alpha E_{\alpha-\beta,1+\alpha}\left(-\left(\frac{t}{t_0}\right)^{\alpha-\beta}\right) \quad \text{(S.49)}$$

$$\omega_{\text{GKV},x} \overset{t_0=1\,\text{s}}{=} \left(\frac{\sin\left(\frac{\pi\beta}{2}\right)-\cos\left(\frac{\pi\beta}{2}\right)}{\cos\left(\frac{\pi\alpha}{2}\right)-\sin\left(\frac{\pi\alpha}{2}\right)}\right)^{1/(\alpha-\beta)} \quad \text{(S.50)}$$

with the Mittag-Leffler function $E_{\kappa,\mu}(x) = \sum_{n=0}^{\infty}\frac{x^n}{\Gamma(\kappa n+\mu)}$.

In Supp. Fig. S.10† selected cases of the tuple (α, β) are shown for $G_0 = 1\,\text{Pa}$ and $t_0 = 1\,\text{s}$ for the GM and GKV and for the SFE the power exponent α is used. Without loss of generality since α and β are interchangeable, in the following the case $\alpha > \beta$ is regarded. Figure S.10A shows a case where $\alpha+\beta = 1$ and therefore $\omega_{\text{GKV},x} = \omega_{\text{GM},x} = \frac{1}{t_0} = 1\,\frac{\text{rad}}{\text{s}}$. For frequencies $\omega \ll \omega_x$ the complex modulus obtained with the GM behaves as ω^α, while with the GKV it behaves as ω^β. Oppositely, for frequencies $[\omega \gg \omega_x : G^* \sim \omega^\beta]$ for the GM and $[\omega \gg \omega_x : G^* \sim \omega^\alpha]$ for the GKV. Note that for $\omega < \omega_x$ the imaginary part is higher than the real part for the GM and vice versa for the GKV and accordingly, for $\omega > \omega_x$ the real part greater than the imaginary part for the GM and vice versa for the GKV. The complex shear modulus cannot be described with one power exponent close to the crossover frequency. Figure S.10B shows a case with $\beta = 0$ and $\alpha+\beta \neq 1$. Here, the crossover frequency is not the same for the GM and the GKV. Also, for frequencies $[\omega \ll \omega_x : G^*_{\text{GM}} \sim \omega^\alpha, G'_{\text{GKV}} \sim \text{const.}, G''_{\text{GKV}} \sim \omega^\alpha]$ while $[\omega \gg \omega_x : G'_{\text{GM}} \sim \text{const.}, G''_{\text{GM}} \sim \omega^{-\alpha}, G^*_{\text{GKV}} \sim \omega^\alpha]$. In Fig. S.10C $\alpha = 0.2$ and $\beta = 0$ there is no crossover frequency. Other than that, the moduli behave as in the case in Fig. S.10B. For power exponents equal to zero (Fig. S.10D) the imaginary part vanishes for the SFE, GM and GKV, while the real part is constant. Note that the constant G_0 is different for SFE, GM and GKV. The value of $G_{\text{GM},0} = 0.5 \cdot G_{\text{SFE},0}$ and $G_{\text{GKV},0} = 2 \cdot G_{\text{SFE},0}$, therefore to compare the absolute value of the constant G_0 between the different models the scaling factor has to be considered. Figure S.10E shows the case where $\alpha = \beta = 0.5$. In this case, the real part equals the imaginary part and they behave as $\omega^\alpha = \omega^\beta$. The difference between each model is due to the difference in the definition of the constant G_0. In Fig. S.10F the case $\beta > \alpha$ is shown with the values of α, β exchanged with each other compared to the case in Fig. S.10A. Indeed, there is no difference in the GM and GKV model in Fig. S.10A and Fig. S.10F and the power exponents are interchangeable. The difference seen in the SFE model is due to the choice of α being the sole power exponent in the model.

Supplementary Figure S.11† shows the fit to a real data set with the different models. Due to the existence of a crossover frequency, the SFE model was used separately for the real and imaginary part of the complex shear modulus.

Bead immersion half-angle correction

The complex shear modulus is defined as the ratio of the stress to strain. However, during the experiment only the force and the displacement of the bead with radius R is measured. Here, to estimate the stress and strain the complex modulus is calculated with a correction $f(\theta)$ based on the bead immersion half-angle θ into the cell

$$\tilde{G}(\omega) = \frac{\tilde{F}(\omega)}{\tilde{z}(\omega)}\frac{1}{6\pi R f(\theta)} \quad \text{(S.51)}$$

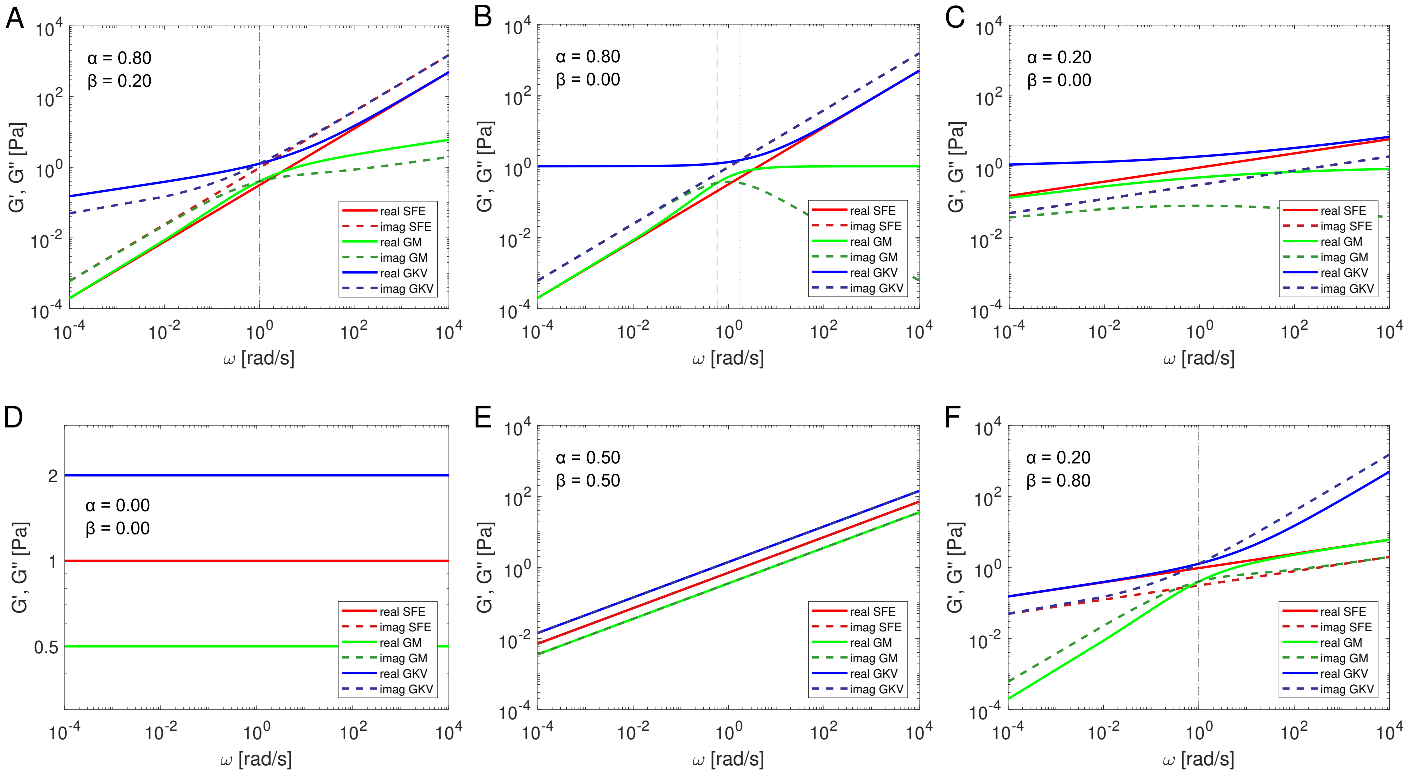

Fig. S.10 Calculated moduli for the single fractional element (SFE) model (red), Generalized Maxwell (GM) model (green) and Generalized Kelvin-Voigt (GKV) model (blue) for selected cases of the power exponents. Solid lines represent the real part of the complex shear modulus and dashed lines the imaginary part. The vertical black dashed line represents the crossover frequency of the GM model and the vertical black dotted line the crossover frequency of the GKV model. (A) Case for $\alpha+\beta=1$ and $\alpha=0.8$, $\beta=0.2$. (B) Case for $\alpha+\beta\neq 1$ and $\alpha=0.8$, $\beta=0$. (C) Case for $\alpha<0.5$ and $\alpha=0.2$, $\beta=0$. (D) Case for $\alpha=\beta=0$. (E) Case for $\alpha=\beta=0.5$. (F) Case for $\alpha<\beta$ and $\alpha=0.2$, $\beta=0.8$.

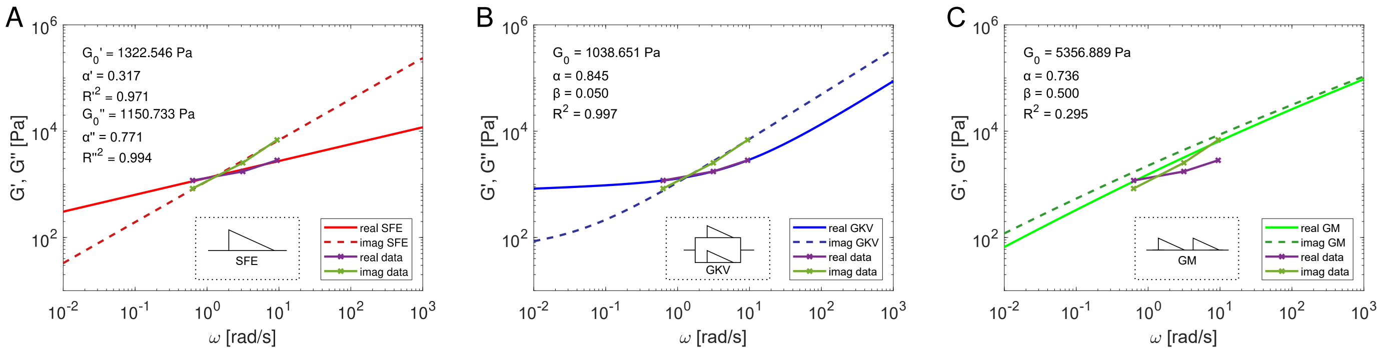

Fig. S.11 Fit of the moduli with the SFE (A, red), GKV (B, blue) and GM (C, green). Solid lines represent the real part of the complex shear modulus and dashed lines the imaginary part. For the SFE the real and imaginary part are evaluated separately. For the GKV and GM the real and imaginary part are evaluated simultaneously. Inset shows the schematic representation of the respective models after the design by Schiessel *et al.*[5]

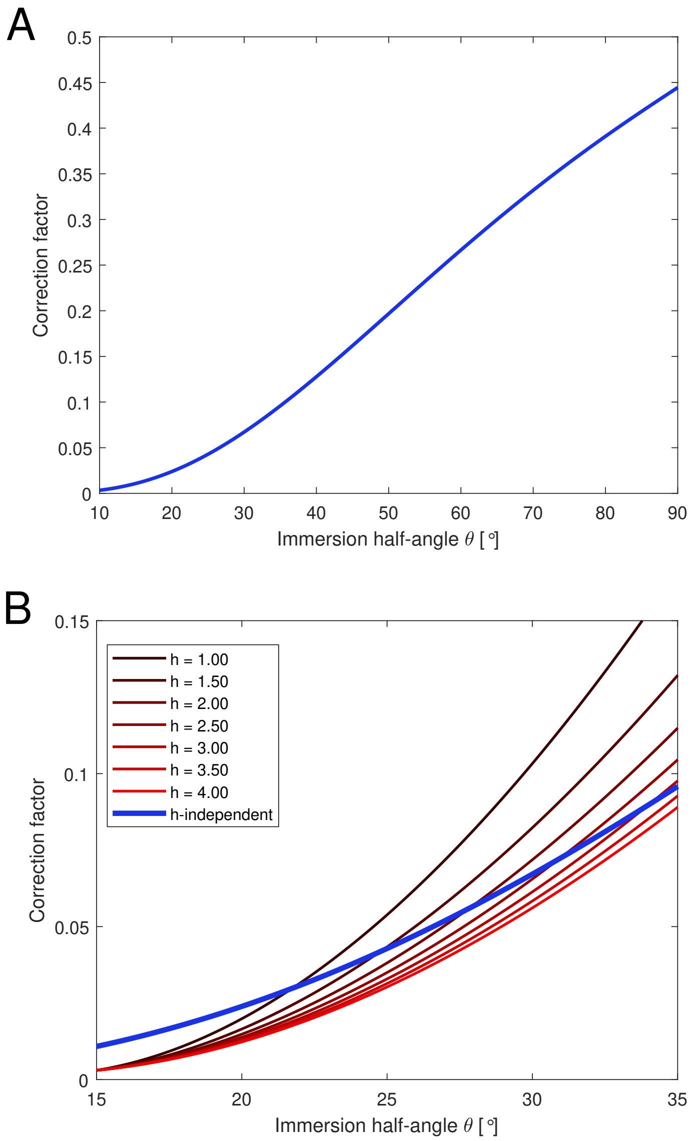

Fig. S.12 Different correction factors to calculate the complex modulus with Eq. S.51. (A) Correction factor $f_1(\theta)$ in the range of $[10°, 90°]$ obtained from [7] which is also used in the analysis. (B) Correction factor $f_2\left(\theta, \frac{h}{2R}\right)$ obtained from [9] which also takes the cell height h into consideration as indicated in red colors and the correction factor $f_1(\theta)$ (blue).

with the correction [7]

$$f_1(\theta) = \left(\frac{9}{4\sin(\theta)} + \frac{3\cos(\theta)}{2\sin^3(\theta)}\right)^{-1}. \tag{S.52}$$

Another immersion half-angle based correction which also takes the cell height h at the location of the bead into consideration is given by [9]

$$f_2\left(\theta, \frac{h}{2R}\right) = A(\theta) + \frac{B(\theta) \cdot 2R}{h}, \tag{S.53}$$

$$A(\theta) = A_0 + A_1\theta + A_2\theta^2 + A_3\theta^3, \tag{S.54}$$

$$B(\theta) = B_0 + B_1\theta + B_2\theta^2 + B_3\theta^3 \tag{S.55}$$

with the constant coefficients shown in Table S.11. The correction factor is also shown in Supp. Fig. S.12† for both corrections. Due to the fact that the cell height at the location of the bead was unknown during the experiment, the correction $f_1(\theta)$ was used for the analysis. However, the height of HUVECs usually does not exceed $3\,\mu$m at the cell body and is $\geq 1\,\mu$m at cell periphery. [10] The difference between the other correction ($f_2\left(\theta, \frac{h}{2R}\right)$) can be considered as a possible error range due to the estimation of the stress and strain.

	A_0, B_0	$A_1, B_1\left[\frac{1}{\text{rad}}\right]$	$A_2/B_2\left[\frac{1}{\text{rad}^2}\right]$	$A_3, B_3\left[\frac{1}{\text{rad}^3}\right]$
A	$2.321 \cdot 10^{-2}$	$-2.054 \cdot 10^{-1}$	$5.250 \cdot 10^{-1}$	$-1.338 \cdot 10^{-1}$
B	$4.788 \cdot 10^{-3}$	$-4.314 \cdot 10^{-2}$	$1.020 \cdot 10^{-1}$	$-2.698 \cdot 10^{-2}$

Table S.11 Values of constants to calculate the polynomials of the correction of the complex modulus [9]

Comparison to optical tweezers

Phagocytosed $1\,\mu$m polystyrene beads in HUVECs were used to measure the viscoelastic properties of HUVECs with optical tweezers. The measurement time for one bead was about 100 s. Searching the next bead and setting up the required conditions for the measurement required on average 6 min. Thus, measuring 15 beads would take about 115 min and these results would correspond to different times as the measurements are shifted. Using the AFS, however, we could on average measure 15 beads simultaneously, drastically increasing the throughput while also obtaining property values every 100 s for each bead at the same time.

As used in our results with the AFS, the results of optical tweezers measured on HUVECs can also be described using the generalized Kelvin-Voigt (GKV) model (Eq. S.27). Since we could only measure phagocytosed beads inside HUVECs, it is expected that the obtained results differ from the AFS where we had measured the cortex. However, the GKV model can well describe the measured data, see Supp. Fig. S.13†.

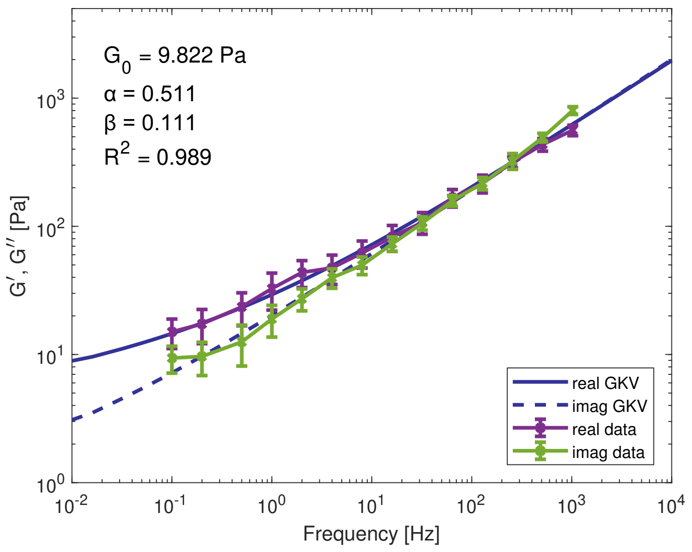

Fig. S.13 Microrheology on HUVECs with the optical tweezers using phagocytosed beads. The data is described by the generalized Kelvin-Voigt model.

Dynamics of the shear modulus with the GKV model

Images of the cells with beads during the control measurement are shown in Supp. Fig. S.14†. Figure 7E shows the obtained parameter G_0 using the GKV model. To test for a significant difference the obtained values are grouped into the time intervals in which the conditions had changed, e.g. a higher flow with cyto B insertion or the washing out process. The significance tests are shown in Supp. Fig. S.15A†. The fit qualities with $R^2 > 0.7$ are shown in Fig. 7E. Supplementary Figure S.15B† shows the fit quality of each obtained data value. Indeed, the fit quality is mainly above $R^2 = 0.9$, although a fit quality of $R^2 = 0.7$ would still be valid for a simultaneous fit of the real and imaginary part of the complex shear modulus.

Data post-processing and analysis

The obtained microrheological data has been post-processed using our software *Kitsune* to obtain correct values for the viscoelasticity. The software filters for inter-particle errors in the tracking, corrects for drift in the long time measurements and includes the local distribution of the conversion factor. Details about the analysis are shown in the next section. Briefly, the software corrects for the following three main problems:

a) During the constant medium flow, other particles can interfere with the tracking of the beads that will cause erroneous values in the (x, y, z)-position data. Typically, these erroneous values only appear for a short time, i.e. one or two data points, due to the relatively high flow rate compared to the sampling frequency. This problem is addressed by replacing the erroneous value with the median value of data points temporally close to it.

b) The long measurement time may introduce mechanical drifts or allows drifts caused by active, but slow, cell movements over time. The drift is corrected by obtaining the drift profile with segmented, continuous fits of polynomial functions of second order and subtracting them from the data. This correction can be performed because the cell movement and activity themselves are not of interest in this case.

c) The lateral inhomogeneity of the force distribution inside the field of view will cause a wrong assignment of the actual force acting on the beads. This can be corrected by assigning a conversion factor of the amplitude to force for each bead according to their current lateral position with the obtained spatial map from the SFC, as described in the next section.

Detailed data post-processing and analysis

The relevant, measured data are the positional data in x, y, z, the amplitude corresponding to the exerted acoustic force F and the sampling time t. A complete data set consists of the measured data in the full duration of acquisition, e.g. a duration of 3700 s. The following shows the automatized steps of the analysis using the self-written MATLAB software *Kitsune (version 0.98)*.

- Find the start and end time of the mOsc corresponding to the data with force type $F_t = 5$ in the modified LabVIEW software. (Full force span)
- Segment the full force span in sections of 500 s evaluation time intervals in 100 s time steps.
- Analyze each of the sections separately with the following steps.
- Get the mean x, y-position during the analyzed section and calculate the force value according to the conversion factor from the calibration map.
- Subtract the force values of the section with its mean to avoid the errors from the zero-padding in a later step. This is valid because the oscillation is of interest and not the initial force step.
- Clean the z-data from tracking errors, e.g. due to particles interfering with the tracking's region of interest (RoI) during flow.
  - Segment the section into 1 s sections. (Error-Section)
  - Calculate the median of the Error-Section. (Local-Median)
  - Find data points within the Error-Section and replace them with the Local-Median if their difference with the Local-Median is greater than 500 nm.
  - Calculate the median of the whole section. (Global-Median)
  - Find data points within the Error-Section and replace them with the Global-Median if their difference with the Global-Median is greater than 10000 nm.
- Correct the z-drift using the continuous poly2 subtraction. (Note that it is not necessarily smooth, but only continuous.)
  - Segment the section into 25 s sections and the final section with at least 3 data points else it is filled with the last value. (Drift-Sections)

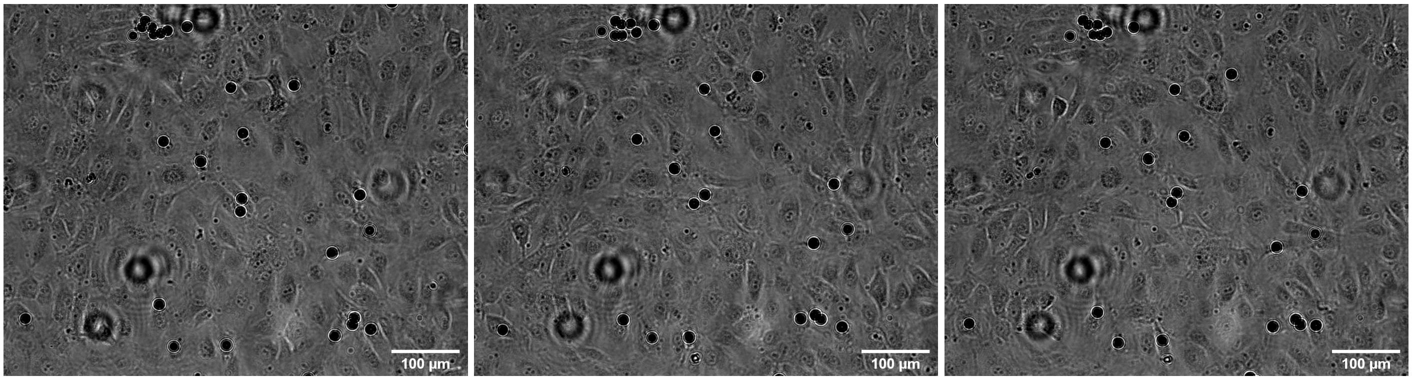

Fig. S.14 (Left) Image of the cells with beads during the control measurement at the time point $500\,\mathrm{s}$, (Center) at the time point $1500\,\mathrm{s}$ when the flow is increased, (Right) at the time point $3000\,\mathrm{s}$ at initial flow rate. Images are slightly out of focus for the bead tracking and to increase contrast. Additionally, images have been edited to further increase contrast. Scale bar is $100\,\mu\mathrm{m}$.

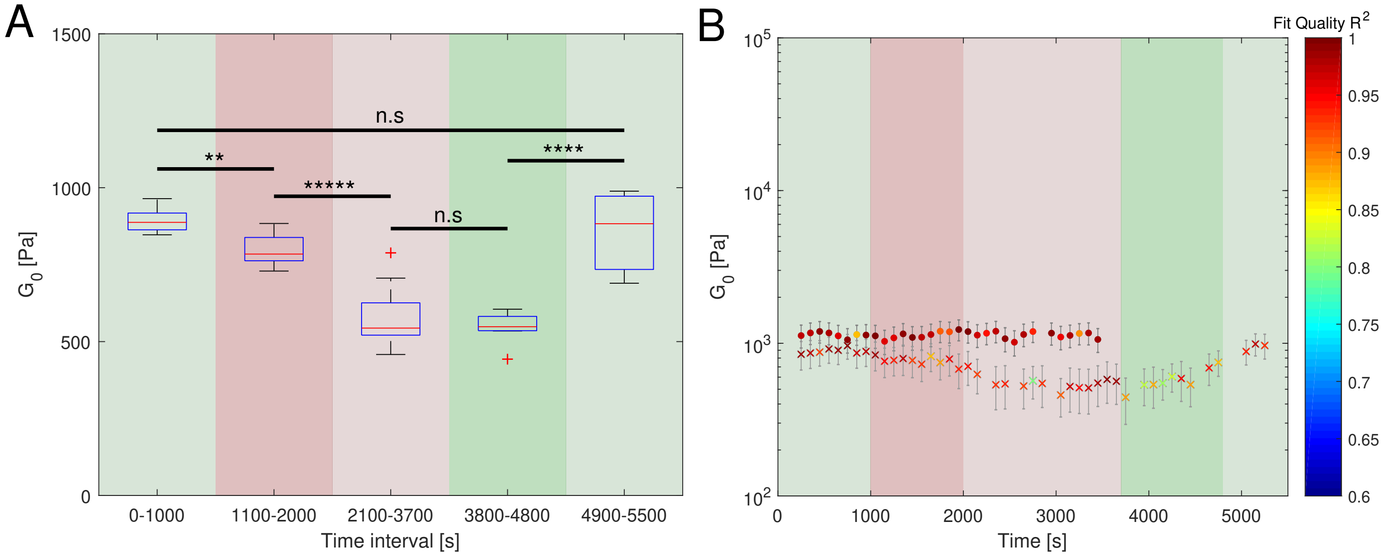

Fig. S.15 (A) Significance tests between different time intervals and conditions for the values of G_0 obtained from the GKV model for the cyto B experiment. The significance star * represents $p \leq 0.05$, $^{**}: p \leq 0.01$, $^{***}: p \leq 0.001$, $^{****}: p \leq 0.0001$, $^{*****}: p \leq 0.00001$ and n.s : not significant, using a two-sample t-test. (B) Representation of Fig. 7E with the fit quality R^2 for each obtained data value.

- Create the Vandermonde Matrix for a polynomial of 2nd order ($n = 2$).
- Constraint the fit: The starting point has to be the ending point of the previous Drift-Section; for the first Drift-Section the starting point of the data is also its starting point.
- Fit the data using lsqlin and polyval which are in-built functions of MATLAB.
- Append all Drift-Sections to a data set. (Drift-Correction)
- Subtract the section with the Drift-Correction.

• Filter out sections (after the correction from above) that have high errors, e.g. a particle interfered with the RoI for too long or the tracked particle is no longer being tracked.

- Calculate the standard deviation (std) of the z-position in the section.
- Set the results to NaN and stop the analysis if at least one of the following criteria are fulfilled:
  1. $\mathrm{std}(z) > 1000\,\mathrm{nm}$
  2. $\mathrm{std}(z) == 0$
  3. $\mathrm{abs}(\mathrm{median}(z)) <= 10^{-8}\,\mathrm{nm}$
  4. $\mathrm{abs}(\mathrm{median}(\mathrm{abs}(z)) - \mathrm{mean}(\mathrm{abs}(z))) > 200\,\mathrm{nm}$
  5. $\mathrm{abs}(\mathrm{median}(z)) > 1000\,\mathrm{nm}$
  6. $\mathrm{abs}(\mathrm{mean}(z)) > 1000\,\mathrm{nm}$
  7. $\mathrm{abs}(\mathrm{abs}(\mathrm{mean}(\mathrm{abs}(z(\text{1st half})))) - \mathrm{abs}(\mathrm{mean}(\mathrm{abs}(z(\text{2nd half}))))) > 50\,\mathrm{nm}$

• Calculate the Discrete-Time Fourier Transformation (DTFT) of z and F.

- Calculate the mean time steps between each data point inside the section due to possible sampling time errors.
- From the mean time step, calculate the mean sampling frequency.
- If the length of the data points inside the section is odd, remove the last data point to make it an even length.
- Calculate the DTFT by zero-padding the data until the length is 100-times the data length.
- Multiply the DTFT by the mean time step and by 2 for the normalization and due to the fact of considering the positive frequency only.

• Find the peaks of the absolute of the DTFT.

- Get the maximum of the DTFT within a 0.01 Hz interval around the estimated driving frequencies.

• Calculate the complex modulus G^* using Eq. S.51 with the peaks of the DTFT of z and the peaks of the DTFT of the force and the determined angle, here $\theta = 28.73\,°$.

• The mean absolute, real and imaginary part of G^* of all n beads is calculated with the standard error $\Delta G^* = \frac{\sigma}{\sqrt{n}}$ and the standard deviation σ.

• The errors of the ratio of real and imaginary part as well as the phase is calculated with error propagation.

• The fit parameters (G_0, α) of the SFE (power) model is obtained by fitting to the logarithm of the Eq. S.20† for real and imaginary part separately by minimizing the value $1 - R^2$ for each fit, respectively. The logarithmic value of G_0 is transformed appropriately. The error is obtained with $n_{\mathrm{boot}} = 100$ bootstrap samples. The values are set to NaN if $R^2 < 0.9$.

• The fit parameters (G_0, α, β) of the GKV model is obtained by fitting the real part of the complex shear modulus to the real part of Eq. S.27† and the imaginary part to the imaginary part of Eq. S.27†. The sum of $1 - R^2$ of each fit is then minimized. The error is obtained with $n_{\mathrm{boot}} = 100$ bootstrap samples. The values are set to NaN if $R^2 < 0.7$.

SFC Protocol

This protocol is used to perform the Stokes Force Calibration (SFC) on Acoustic Force Spectroscopy (AFS) chips. It also serves as a guide. It is part of the Supplemental Information of the publication *Multi-oscillation microrheology via Acoustic Force Spectroscopy enables frequency-dependent measurements on endothelial cells at high-throughput*. The calibration method is described in the main text of the publication and the recommended software *Kitsune* to analyze the SFC data is available on github `https://github.com/A141-User/Acoustic-Kitsune`. This protocol is for the G2 AFS chips; other generations may differ.

Conditions of the main experiment

The conditions of the main experiment has to be set beforehand. Firstly, to ensure that the AFS can perform under the conditions and, secondly, to perform the SFC under the same conditions due to the dependency of the force on the respective conditions.
Conditions:

- Temperature ($T < 40\,°$)
- Medium (liquid during experiment)
- Bead size (preferably $0.5\,\mu\text{m} < d < 20\,\mu\text{m}$)
- Bead type (e.g. polystyrene, silica, etc.)
- Desired force range (pN-nN)
- (optional) to think about:
  - force type, e.g. constant force, force ramp, etc.
  - length of force application

Preparation

Prior to a successful calibration there are still a few aspects to be taken care of.

- This calibration method is based on video-capturing, meaning that a camera with a sampling rate of $f_s > 30\,\text{Hz}$ is recommended.
- The surface of the AFS fluid chamber is made of glass. The beads in combination of the medium may bind to the surface, e.g. due to charge. Therefore, the glass or the beads can be coated accordingly to prevent specific binding on the surface.
- The bottom of the chip should be cleaned to prevent dirt that can disturb the tracking and may also alter the force values.
- The inside of the fluid chamber should be cleaned, e.g. with ethanol or standard bleach (e.g. A1727 Sigma-Aldrich) and thoroughly washed out afterwards.
- The position of the AFS chip is fixed and can only be moved in a controlled manner to ensure to measure at the same field of view (FoV).

Procedure

After meeting the conditions of the main experiments and a successful preparation the SFC can be performed. The temperature, medium and beads mentioned here are the ones of the main experiment for which the following calibration is performed.

1. set the temperature (wait at least 1 min)
2. suspend the beads in the medium (bead suspension)
3. insert the bead suspension into the chip
4. let the beads fall to the ground by gravity

Prior to actually measuring the beads, it might be more feasible to just observe the beads in the bright-field image when applying a low amplitude first to quickly scan through the chip for usable FoVs. Find the desired FoV and make sure to be able to find the same FoV even after unmounting the chip from the microscope.

It is recommended to use the resonance frequency because the ratio of the axial force to the lateral force is typically higher at the resonance frequency. The following steps are to obtain the resonance frequency.

1. track all single beads
2. create a look-up-table (LUT) in the range greater than the first z-node, typically $20\,\mu\text{m}$ for a specific type of chips
3. measure all beads (here, if possible with a moving region of interest (RoI))
4. as a first estimation, use the recommended frequency saved inside the chip
5. apply a constant pressure amplitude for 1 s
6. change the frequency by $\Delta f = 0.01\,\text{MHz}$ (in both directions)
7. after the bead is at the ground again, repeat the same constant pressure amplitude for 1 s
8. repeat steps 6. and 7. until the resonance frequency is found which can be seen by the measured z-position (fastest rise to the z-node) or a quick analysis using *Kitsune* (highest force)
9. to prepare the measurement to obtain the conversion factor which requires several constant pressure amplitude values, check for the highest possible amplitude at which the z-position of the bead can be sufficiently recorded by the camera

Now, the resonance frequency is found and the SFC can be performed to obtain the spatial calibration map of the FoV showing the conversion factors using the following steps.

1. insert new beads
2. wait until they are on the ground
3. track, create LUT and measure the positional data of the beads (do not use a moving RoI)
4. apply a low amplitude for 1 s

5. wait until they are on the ground
6. apply a higher amplitude for 1 s
7. repeat steps 5. and 6. until a desired amount of amplitudes are measured (preferably 4 different amplitudes smaller than the highest possible to capture)
8. end measurement
9. repeat steps 1-8 until a desired amount of beads are measured, i.e. the measured beads are well-distributed throughout the whole FoV (which can be quickly checked using *Kitsune*'s "Bead distribution")

The experimental procedure is completed and the xyz-positional data of every bead has been measured. For every bead, the z-position has to be evaluated during the force application. The lateral location of each bead is known and the conversion factor is assigned to each bead to obtain the distribution of the conversion factor. In order to obtain the calibration map of the conversion factors, the distribution has to be interpolated. The analysis can be performed using the steps shown in the main text of the publication or using *Kitsune*.

Further steps

After obtaining the calibration map for one FoV at one specific condition, the calibration is basically successful. A few other steps can be performed, as listed in the following.

- find other FoVs and obtain the maps (calibration), make sure to assign the maps to the FoV accordingly
- calibrate the FoV at different conditions, such as different temperatures
- measure the temperature change inside the flow chamber during the SFC force with an external temperature sensor if the temperature sensor inside the chip holder cannot measure the temperature inside the flow chamber
- measure the temperature change inside the flow chamber during force application of the main experiment
- obtain the temperature dependency of the force at the FoV if the temperature change is significantly high
- perform the main experiment
- calibrate the same FoV at the same condition again after a while to account for degradation
- calibrate other AFS chips

Additional notes

- The analysis software *Kitsune* is available on request and is based on Matlab R2017 (The MathWorks). It is written for data generated by a modified version of the Generation 2 AFS LabVIEW tracking software. However, it can also analyze the standard version.
- The density of the bead has to be greater than the density of the medium under the conditions.
- If a force type other than the constant force or force ramp is needed, the LabVIEW software may have to be modified.
- If a high force amplitude is applied, the change of the temperature inside the fluid chamber during force application should be measured.

References in the ESI

2 M. Settnes and H. Bruus, *Physical Review E*, 2012, **85**, 016327.

3 H. Bruus, *Lab on a Chip*, 2012, **12**, 1014.

4 D. Kamsma, R. Creyghton, G. Sitters, G. J. Wuite and E. J. Peterman, *Methods*, 2016, **105**, 26–33.

5 H. Schiessel, R. Metzler, A. Blumen and T. F. Nonnenmacher, *Journal of Physics A: Mathematical and General*, 1995, **28**, 6567–6584.

6 P. Kollmannsberger, C. T. Mierke and B. Fabry, *Soft Matter*, 2011, **7**, 3127–3132.

7 M. Balland, N. Desprat, D. Icard, S. Féréol, A. Asnacios, J. Browaeys, S. Hénon and F. Gallet, *Physical Review E*, 2006, **74**, 021911.

8 F. Ziemann, J. Rädler and E. Sackmann, *Biophysical Journal*, 1994, **66**, 2210–2216.

9 A. Kamgoué, J. Ohayon and P. Tracqui, *Journal of Biomechanical Engineering*, 2007, **129**, 523–530.

10 I. Alesutan, J. Seifert, T. Pakladok, J. Rheinlaender, A. Lebedeva, S. T. Towhid, C. Stournaras, J. Voelkl, T. E. Schäffer and F. Lang, *Cellular Physiology and Biochemistry*, 2013, **32**, 728–742.
